# Recent advances in therapeutic strategies for triple-negative breast cancer

**DOI:** 10.1186/s13045-022-01341-0

**Published:** 2022-08-29

**Authors:** Yun Li, Huajun Zhang, Yulia Merkher, Lin Chen, Na Liu, Sergey Leonov, Yongheng Chen

**Affiliations:** 1 Department of Oncology, NHC Key Laboratory of Cancer Proteomics, Laboratory of Structural Biology, Xiangya Hospital, Central South University, Changsha, 410008 Hunan China; 2 School of Biological and Medical Physics, Moscow Institute of Physics and Technology, Dolgoprudny, Moscow Region, Russia 141700; 3 Department of Endocrinology, Xiangya Hospital, Central South University, Changsha, 410008 Hunan China; 4 Institute of Cell Biophysics, Russian Academy of Sciences, Pushchino, Russia 142290; 5 National Clinical Research Center for Geriatric Disorders, Xiangya Hospital, Central South University, Changsha, 410008 Hunan China

**Keywords:** Triple-negative breast cancer, Targeted therapy, Immunotherapy, Combination therapy

## Abstract

Triple-negative breast cancer (TNBC) is the most malignant subtype of breast cancer (BC) with a poor prognosis. Current treatment options are limited to surgery, adjuvant chemotherapy and radiotherapy; however, a proportion of patients have missed the surgical window at the time of diagnosis. TNBC is a highly heterogeneous cancer with specific mutations and aberrant activation of signaling pathways. Hence, targeted therapies, such as those targeting DNA repair pathways, androgen receptor signaling pathways, and kinases, represent promising treatment options against TNBC. In addition, immunotherapy has also been demonstrated to improve overall survival and response in TNBC. In this review, we summarize recent key advances in therapeutic strategies based on molecular subtypes in TNBC.

## Introduction

Breast cancer (BC) is the most commonly diagnosed cancer among women and the second leading cause of cancer-related mortality worldwide [1]. Based on molecular markers, including estrogen receptor (ER), progesterone receptor (PR), and human epidermal growth factor receptor 2 (HER2), BC is categorized into three major subtypes: hormone receptor (HR)-positive, HER2-positive, and triple-negative breast cancer (TNBC). TNBC accounts for approximately 15% to 20% of all breast carcinomas [2]. Compared with HR-positive BCs, TNBC has a worse prognosis. Greater than 50% of patients experience a relapse in the first 3 to 5 years after diagnosis [3], and the median overall survival (OS) based on current therapies is 10.2 months [4].

Patients with TNBC do not benefit from established endocrine or HER2-targeted drugs due to a lack of related receptor markers. Therefore, the standard of care for nonsurgical TNBC remains nonspecific chemotherapy. TNBC is the subtype with the best response to standard chemotherapy regimens, such as taxanes or anthracyclines. However, less than 30% of patients with TNBC achieve a complete response, and the recurrence and mortality rates remain higher than those of non-TNBC subtypes. Although TNBC is a clinical tumor entity, whole-genome sequencing studies have shown extensive intertumoral and intratumor molecular heterogeneity and have facilitated classifications of tumor subtypes [2]. The most recognized subtypes were Lehmann's six clusters in 2011, which include two basal-like (BL1 and BL2), immunomodulatory (IM), luminal androgen receptor (LAR), mesenchymal (M), and mesenchymal-stem-like (MSL) subtypes [5]. Previously, a few small-molecule inhibitors, bromodomain and extra-terminal domain inhibitors, have demonstrated efficacy in TNBC. However, rapid resistance to these drugs develops via multiple mechanisms [6]. Therefore, determining the molecular characteristics of TNBC, targeting specific changes in the internal and external tumor environment, and developing new treatment regimens represent demands in this field that must be urgently met. Considering the malignancy, heterogeneity, and drug resistance, multiple targeted therapeutic approaches and combinations of regimens are essential to improve the outcome of TNBC. In this review, we summarize some promising approaches to address the unmet needs of TNBC subtypes based on integrated omics data for recent treatment progress.

## Molecular subtypes and characteristics of triple-negative breast cancer

To avoid blindly developing therapeutic strategies, identifying the complex TNBC subtypes and molecular hallmarks is necessary given that these features are closely linked with clinical outcomes, for example, response to chemotherapy, the pattern of recurrence, and prognosis. Different approaches, including somatic DNA mutation, copy number aberrations, gene expression profiling, and immune metagene information, were applied to analyze TNBCs as a highly diverse group of cancers.

Initially, six clusters were distinguished from 21 breast cancer datasets by Lehmann in 2011 [5]. Gene Ontology analysis showed that BL1 and BL2 subtypes were involved in the DNA damage response and cell cycle genes, preferentially responding to cisplatin. The LAR subtype exhibits high expression of genes associated with increased androgen receptor (AR) signaling and response to AR antagonism. The M and MSL subtypes were manifested by increased expression of genes involved in cell differentiation and growth factor pathways. The sensitivity of these subtypes to the phosphoinositol-3 kinase (PI3K)/mammalian target of rapamycin (mTOR) inhibitor and the ABL/SRC inhibitor was demonstrated in cell models. The IM cluster was enriched in multiple immune signaling pathways. This work made outstanding contributions to shedding light on drug design and clinical therapy. In 2016, TNBC was subdivided into four groups (BL1, BL2, M, and LAR) for the selection of neoadjuvant chemotherapy (NAC) [7]. According to a previous description, the IM and MSL subtypes originate from infiltrating lymphocytes and tumor-associated stromal cells, respectively. In the revised classification, groups differed in response to chemotherapy, local and distant disease progression, and prognosis. Combined analysis showed that the highest and lowest pathological complete response (pCR) rates were 41% for BL1 patients and 18% for BL2 patients administered similar NAC regimens [7]. Burstein and colleagues also sought to redefine four clusters, including LAR, mesenchymal, basal-like immune-suppressed (BLIS), and basal-like immune activated. The BLIS cluster had the worst prognosis in terms of disease-free survival (DFS), suggesting the important role of the immune system in TNBC [8]. Yi-Zhou Jiang et al. classified TNBCs into 4 subtypes, including LAR, immunomodulatory, basal-like immune-suppressed, and mesenchymal-like, based on RNA sequencing, exome sequencing, and copy number array analyses of TNBC cases in China in 2019 [9]. In addition, these researchers found increased frequencies of PIK3CA mutations and LAR subtypes compared with that noted in previous data from The Cancer Genome Atlas (TCGA), offering potential clinical management with subtype-specific and molecular targeted therapies. Immune metagene information clustered TNBC into three subtypes: C1 (LAR), C2 (BL with a low immune response but high M2-like macrophages), and C3 (BL with a high immune response but low M2-like macrophages). C3 patients had significantly better event-free survival than C2 patients [10].

Additionally, molecular alterations were assessed to explore various potential targets for TNBC treatment. It is worth mentioning that a deficiency in homologous recombination, which is partly associated with the loss of breast cancer susceptibility gene (BRCA) function in BC, is correlated with a good response to cisplatin treatment [11]. In an early phase II clinical trial, patients with BRCA-mutant TNBC showed an overall response rate (ORR) of 80% with single cisplatin therapy [12]. A deficiency in homologous recombination means failure to repair DNA double-strand breaks and damaged DNA replication forks. Therefore, these individuals are also sensitive to poly-adenosine diphosphate [ADP]-ribose polymerase (PARP) inhibitors (PARPi), as PARP is the enzyme that responds to repair DNA single-strand breaks and maintain genome stability.

**Fig. 1:**
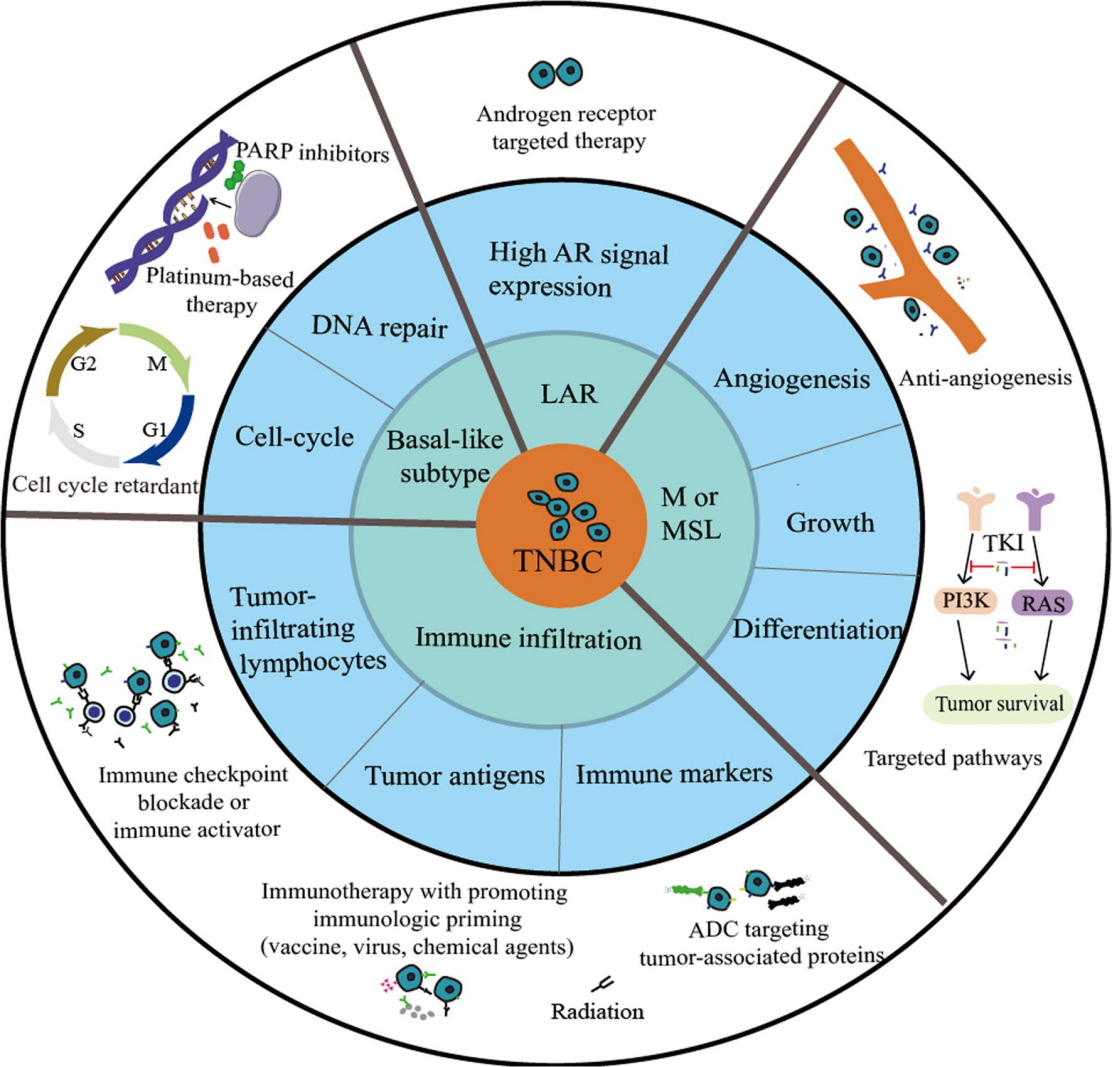
Classification and therapeutic options for TNBC. ADC: antibody‒drug conjugates; AR: androgen receptor; LAR: luminal androgen receptor; M: mesenchymal; MSL: mesenchymal-stem-like; PARP: poly-adenosine diphosphate ribose polymerase; PI3K: phosphoinositol-3 kinase; TKI: tyrosine kinase inhibitor; and TNBC: triple-negative breast cancer

As summarized by Denkert, augmented proliferative activity, increased immune cell infiltration, basal-like and mesenchymal phenotypes, defective homologous recombination partly associated with loss of function of BRCA1, and the androgen receptors overexpression are all distinctive features of TNBC [11]. Therefore, valuable knowledge of subtype characteristics and molecular alterations has shed light on several promising directions, such as molecular-based precise therapies and immunotherapeutic interventions. An overview of the classifications and approaches to treat TNBC is shown in Fig. 1.

## Molecular targeted therapy and potential treatment regimens

**Fig. 2:**
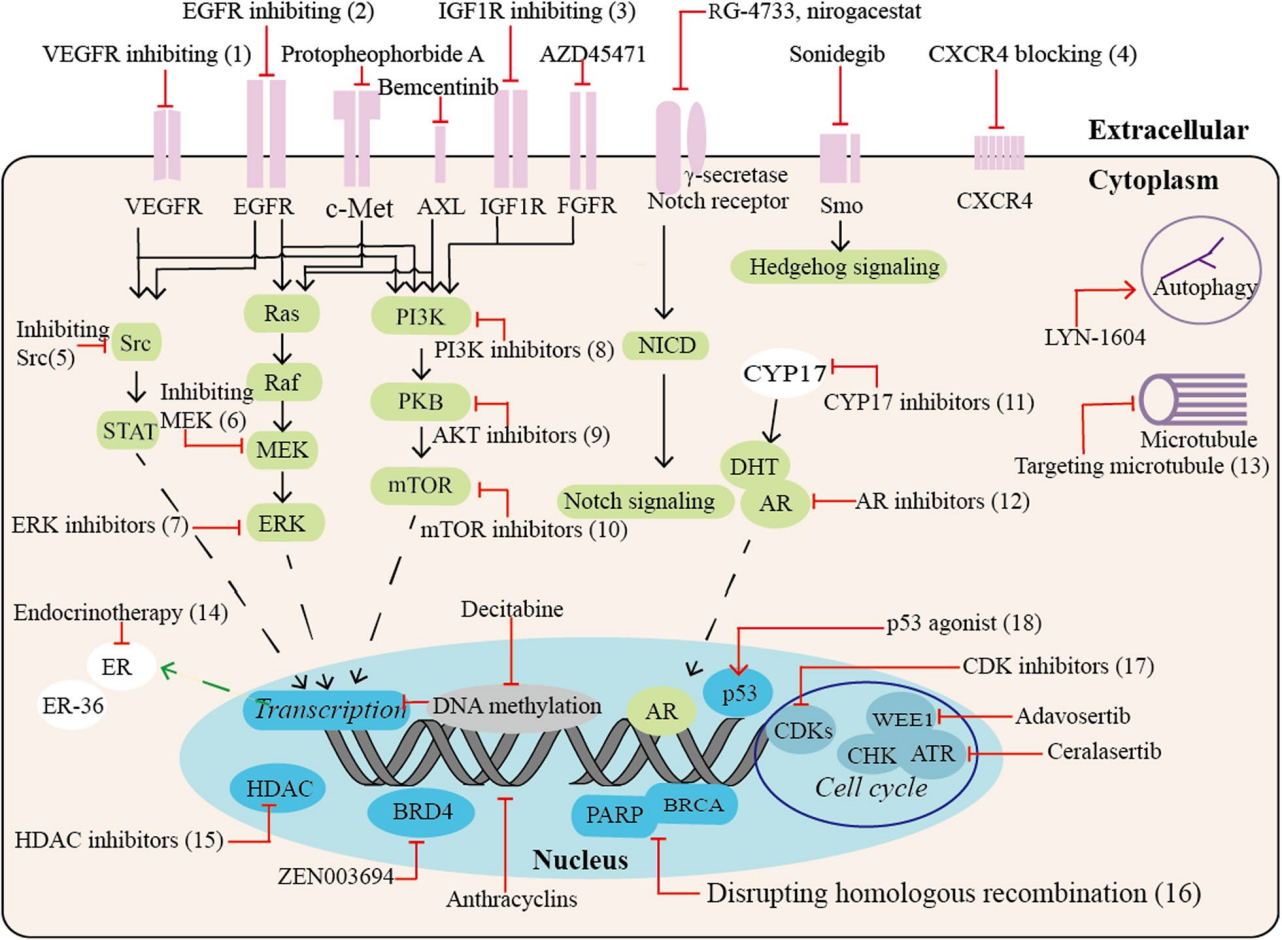
Potential therapeutic targets and appropriate drugs in TNBC. The schematic shows several major abnormal signaling pathways (green), excessive activated receptors (purple), and other key molecules involved in proliferation and progression (blue) in TNBC. Drugs specifically targeting molecules are indicated by red arrows, and the number represents the following agents: (1) VEGFR inhibitors (cediranib, apatinib, lenvatinib) and VEGFR mAb (bevacizumab); (2) EGFR inhibitors (afatinib, gefitinib), EGFR mAbs (nimotuzumab, panitumumab, cetuximab, and SCT200) and ADCs (anti-EGFR-IL-dox and U3-1402); (3) IGF1R blocking drugs (linsitinib, NVP-AEW541, and BMS-754807); (4) CXCR4 antagonists (balixafortide) and CXCR4-binding peptide (DV1); (5) Src inhibitors (dasatinib and BJ-2302); (6) MEK inhibitors (trametinib and binimetinib); (7) ERK inhibitors (BL-EI001 and nifetepimine); (8) PI3K inhibitors (alpelisib and buparlisib); (9) AKT inhibitors (ipatasertib and capivasertib); (10) mTOR inhibitors (everolimus and MLN0128); (11) CYP17 inhibitors (abiraterone acetate and orteronel); (12) AR inhibitors (bicalutamide, enzalutamide, and enobosarm); (13) microtubule stabilizer (taxanes, vincristine, and eribulin); multiple target inhibitors (AMXI-5001 and ixabepilone); and ADCs (mirvetuximab, soravtansine, CX-2009, and SAR566658); (14) endocrinotherapy (tamoxifen and letrozole); (15) HDAC inhibitors (panobinostat, belinostat, chidamide, romidepsin, entinostat, and CUDC-907); (16) PARPi (olaparib, veliparib, talazoparib, niraparib, and rucaparib) and platinum-based agents (cisplatin and carboplatin); (17) CDK inhibitors (trilaciclib, palbociclib, abemaciclib, ribociclib, dinaciclib, and PF-06873600); and (18) p53 agonist (PRMIA-1 and APR-246). ADCs, antibody‒drug conjugates; AR: androgen receptor; AXL: AXL receptor tyrosine kinase; BRCA: breast cancer susceptibility gene; BRD4: bromodomain containing 4; CDK: cyclin-dependent kinases; CXCR4: C-X-C chemokine receptor type 4; CYP17: 17-[α]-hydroxylase/17:20-lyase (CYP17); ER: estrogen receptor; DHT: dihydrotestosterone; EGFR: epidermal growth factor receptor; FGFR: fibroblast growth factor receptor; HDAC: histone deacetylase; IGF1R: type 1 insulin-like growth factor receptor; PARP: poly-adenosine diphosphate ribose polymerase; and VEGFR: vascular endothelial growth factor receptor

Conventional neoadjuvant chemotherapy yielded pCR in approximately 35–45% of patients with TNBC in 2020 [13]. In addition, the majority of patients responsive to standard therapeutic options were limited to the nonmetastatic stage; however, the standard therapeutic options have not significantly changed the overall survival rate. Therefore, analyzing the molecular footprint driving treatment resistance is highlighted. This was a great contribution of Balko and colleagues, who identified the molecular profile of residual TNBC after NAC [14]. Interestingly, they found significant alterations in gene expression after NAC compared with TCGA dataset findings. Of note, great than 90% of the residual patients harbored pathway changes with available targeted treatments, guiding the best selection of targeted therapies. These findings also suggest that combination therapy is likely to solve the problem of incomplete remission. In this molecular profiling study, five key pathways or functional alterations were identified, including cell cycle alterations, PI3K/AKT/mTOR and/or phosphatase and tensin homolog (PTEN) alterations, growth factor receptor amplification, RAS/mitogen-activated protein kinase (MAPK) alterations, and DNA repair alterations. Significantly enriched myeloid cell leukemia-1, myc, and cell cycle-related regulators were found in post-NAC residual TNBC compared with TCGA basal-like tumors. Alterations in PTEN and Janus protein tyrosine kinase 2 (JAK2) were also observed. Furthermore, clinical analysis showed that PTEN alteration predicted a better prognosis for OS, whereas JAK2 amplification and BRCA1 mutation or truncation were regarded as poor prognosis factors [2]. This categorical molecular profile has led to the exploration of rational clinical options for targeted intervention, including cell cycle inhibition, anti-angiogenesis, MAPK and PI3K pathway inhibition, DNA damage response blockade, and their combination. Table 1 summarizes phase II to phase IV clinical trials for molecular targeted therapy based on molecular profiling. Figure 2 shows major therapeutic targets or oncogenic vulnerabilities and their representative agents in TNBC.

**Table 1:** Ongoing phase II–IV trials based on molecularly targeted therapies

Target	Drugs	Design	Register ID	Phase	Status
Cell cycle	Trilaciclib	Trilaciclib with gemcitabine and carboplatin	NCT02978716	II	Active
		Trilaciclib	NCT04799249	III	Recruiting
	Etoposide	Etoposide plus anlotinib	NCT04452370	II	Recruiting
	PF-06873600	PF-06873600 plus endocrinotherapy	NCT03519178	II	Recruiting
	Abemaciclib	Abemaciclib	NCT03979508	II	Recruiting
	Prexasertib	Prexasertib plus samotolisib	NCT04032080	II	Recruiting
		Prexasertib	NCT02203513	II	Active
Microtubule dynamics	Eribulin mesylate	Eribulin mesylate	NCT04502680	II	Not yet recruiting
		Eribulin mesylate, apatinib, and camrelizumab	NCT04303741	II	Recruiting
VEGF/VEGFR	Anlotinib	Anlotinib plus etoposide	NCT04452370	II	Recruiting
		Anlotinib and penpulimab plus chemotherapy	NCT04877821	II	Not yet recruiting
	Apatinib	Apatinib combined with paclitaxel and carboplatin	NCT03735082	II	Unknown
		Apatinib plus capecitabine versus capecitabine	NCT03775928	II	Recruiting
		Apatinib combined with paclitaxel	NCT03348098	II	Unknown
		Apatinib combined with albumin paclitaxel, and carboplatin	NCT03650738	II	Unknown
		Apatinib with camrelizumab, and eribulin mesylate	NCT04303741	II	Recruiting
		Vinorelbine with or without apatinib mesylate	NCT03932526	II	Not yet recruiting
	Afatinib	Afatinib with paclitaxel	NCT02511847	II	Unknown
	Lenvatinib	Lenvatinib plus pembrolizumab	NCT03797326	II	Recruiting
	Erlotinib	Erlotinib with neoadjuvant chemotherapy	NCT00491816	II	Unknown
	Famitinib	Famitinib with camrelizumab and nab-paclitaxel	NCT04395989	II	Recruiting
	Pyrotinib	Pyrotinib with capecitabine			
	Bevacizumab	Bevacizumab and nab-paclitaxel			
		Bevacizumab	NCT03577743	II	Completed
		Bevacizumab	NCT00528567	III	Completed
		Bevacizumab with taxane	NCT01094184	IV	Completed
		Bevacizumab, abraxane, and carboplatin	NCT00479674	II	Completed
		Bevacizumab with nab-paclitaxel followed by bevacizumab and erlotinib	NCT00733408	II	Completed
		Bevacizumab together with docetaxel, and carboplatin	NCT01208480	II	Completed
		Bevacizumab, pegylated liposomal doxorubicin, and everolimus	NCT02456857	II	Active, not recruiting
	Bintrafusp alfa	Bintrafusp alfa (M7824)	NCT04489940	II	Recruiting
EGFR	Dasatinib	Dasatinib	NCT02720185	II	Active
	Gefitinib	Gefitinib	NCT01732276	II	Unknown
	Sorafenib	Sorafenib and pemetrexed	NCT02624700	II	Terminated
	Nimotuzumab	Nimotuzumab plus docetaxel, and capecitabine	NCT01939054	II	Unknown
	Panitumumab	Panitumumab, carboplatin, and paclitaxel	NCT02593175	II	Recruiting
			NCT02876107	II	Recruiting
	SCT200	SCT200	NCT03692689	II	Unknown
PI3K/AKT/mTOR	Alpelisib	Alpelisib and nab-paclitaxel	NCT04216472	II	Recruiting
		Alpelisib with nab-paclitaxel	NCT04251533	III	Recruiting
	Buparlisib	Buparlisib plus capecitabine	NCT02000882	II	Completed
	Eganelisib	Eganelisib with front-line regimens	NCT03961698	II	Recruiting
	Sapanisertib	TAK-228 and TAK-117 followed by cisplatin and nab-paclitaxel	NCT03193853	II	Active, not recruiting
	Samotolisib	Samotolisib and prexasertib	NCT04032080	II	Recruiting
	Ipatasertib	Ipatasertib with nontaxane chemotherapy agents	NCT04464174	II	Recruiting
		Ipatasertib with paclitaxel versus placebo with paclitaxel	NCT03337724	III	Active
		Ipatasertib with atezolizumab, and paclitaxel	NCT04177108	III	Active
	Uprosertib	Uprosertib with trametinib	NCT01964924	II	Completed
	Capivasertib	Capivasertib plus paclitaxel or paclitaxel plus placebo	NCT02423603	II	Active, not recruiting
		Capivasertib with paclitaxel versus placebo with paclitaxel	NCT03997123	III	Recruiting
	Everolimus	Everolimus plus cisplatin	NCT01931163	II	Has results
		Everolimus plus carboplatin compared with carboplatin	NCT02531932	II	Recruiting
HDAC	Entinostat	Entinostat with atezolizumab	NCT02708680	II	Unknown
	Chidamide	Chidamide with Cisplatin	NCT04192903	II	Not yet recruiting
Endocrinotherapy	Estradiol	Estradiol	NCT03941730	II	Recruiting
			NCT01083641	II	Terminated
	Crizotinib	Fulvestrant and crizotinib	NCT03620643	II	Recruiting
	Goserelin	Additional goserelin to the neoadjuvant chemotherapy	NCT03444025	II	Not yet recruiting
	Mifepristone	Nab-paclitaxel with or without mifepristone	NCT02788981	II	Recruiting
	Neratinib	Paclitaxel and carboplatin plus neratinib	NCT03812393	II	Recruiting
	Anastrozole	Anastrozole and entinostat	NCT01234532	II	Terminated
		Anastrozole/toremifene	NCT02089854	IV	Unknown
Other targets					
γ-secretase	AL101	AL101	NCT04461600	II	Recruiting
	PF-03084014	PF-03084014	NCT02299635	II	Terminated
AXL kinase	Bemcentinib	Bemcentinib in combination with pembrolizumab	NCT03184558	II	Terminated
Hedgehog pathway	Vismodegib	Additional vismodegib to neoadjuvant chemotherapy	NCT02694224	II	Unknown
CXCL8 and CXCR1/2	Reparixin	Paclitaxel with or without reparixin	NCT02370238	II	Completed
MEK and ERK	Selumetinib	Neoadjuvant chemotherapy docetaxel with or without selumetinib	NCT02685657	II	Unknown

AKT: serine/threonine kinase; CXCL: chemokine (C-X-C motif) ligand; CXCR: C-X-C motif chemokine receptor; EGFR: epidermal growth factor receptor; HDAC: histone deacetylase; mTOR: mammalian target of rapamycin; MEK: MAP kinse-ERK kinase; PIK3: phosphoinositide-3-kinase; and VEGFR: vascular endothelial growth factor receptor

### Cell cycle retardants

Under physiological conditions, the normal cell cycle is tightly regulated by various factors, such as cyclin-dependent kinases (CDKs), cyclin, and CDK inhibitors. However, the G1-S transition is significantly promoted in the tumor cell cycle, as noted in TNBC. In residual TNBC, the expression of CDKs, including CDK1/2, CDK4, and CDK6, is altered. Inhibitors of CDK1/2 cause cell cycle arrest and apoptosis, and CDK4/6 inhibition leads to G1 arrest [15].

#### Gemcitabine

Gemcitabine, a widespread chemotherapeutic agent mainly acting on the G1/S phase, has already been investigated for combination therapy in patients with TNBC. Gemcitabine with carboplatin and trilaciclib (G1T28, a CDK4/6 inhibitor) has been active in NCT02978716 among cases with metastatic TNBC (mTNBC). Preliminary studies have reported that the cell cycle-related inhibitors, palbociclib, abemaciclib, and ribociclib, also achieved promising antitumor activity in breast cancer, and partial CDK inhibitors were approved by the Food and Drug Administration (FDA) for ER^+^ HER2^−^ advanced or metastatic BC.

#### CDK inhibitors

Several preclinical studies have evaluated CDK inhibitors in TNBC with tyrosine kinase inhibitors (TKIs) in vivo and in vitro. Palbociclib, a CDK4/6 inhibitor, together with a second-generation dual mTOR kinase inhibitor MLN0128, has demonstrated a cooperative suppressed tumor growth effect in retinoblastoma (Rb) protein‑expressing TNBC patient-derived tumor xenograft (PDX) tumors, characterized by a suppressed mTOR pathway and G1/S transition [16]. Palbociclib in combination with the novel PI3K/mTOR inhibitor samotolisib (NCT04032080) is active in a phase II clinical study. Another cell cycle-specific antitumor drug, etoposide, in combination with the multitargeted TKI anlotinib, has also been assessed in TNBC patients (NCT04452370).

The therapeutic effect of CDK 4/6 inhibitor on TNBC is closely related to its substrate Rb [16], while Rb protein expression is closely related to AR positivity (> 10%) [17, 18]. According to previous analysis, activation of AR signaling is an important feature of LAR subtype of TNBC [5]. Therefore, CDK inhibitors combined with AR inhibitors are potential combination strategies, such as palbociclib with AR inhibitors (bicalutamide) in AR^+^ mTNBC (NCT02605486) and in metastatic BC (NCT02605486). Besides, ribociclib plus bicalutamide for advanced AR^+^ TNBCs (NCT03090165) and abemaciclib for Rb protein-positive mTNBCs (NCT03130439) have been used. Other novel CDK inhibitors, such as dinaciclib, PF-06873600, and trilaciclib, have been analyzed in clinical trials to assess the antitumor activity of TNBC [19] (https://clinicaltrials.gov/).

#### Microtubule inhibition

Microtubule inhibition is another effective type of chemotherapy that affects mitosis. The following agents are noted: taxane with an anti-microtubule depolymerization effect and a broader antitumor spectrum; vincristine, a plant chemotherapeutic drug with the ability to alter tubulin polymerization equilibrium; and eribulin, a nontaxane microtubule depolymerizing agent that binds to tubulin and microtubules to inhibit proliferation. These drugs are usually not prescribed alone but in combination with other chemotherapy drugs or immunotherapies. An early preclinical study found that eribulin could inhibit the phosphorylation of AKT (also called protein kinase B, PKB). When administered in combination with the mTOR inhibitor everolimus, eribulin synergistically suppressed tumor growth in vitro as well as in orthotopic mouse models, providing a mechanistic foundation for the treatment of refractory TNBC [20]. Currently, several registered clinical trials on eribulin are going (NCT04502680, NCT01372579, and NCT02225470).

There is continuous research to develop new and different types of microtubule inhibitors. Additional research focuses on coupling these toxic microtubule inhibitors with some antibodies into new antibody‒drug conjugates (ADCs), such as mirvetuximab soravtansine, CX-2009, and SAR566658. Newly designed compounds, such as AMXI-5001, combine the characteristics of several small-molecule inhibitors to achieve dual or multiple target functions. AMXI-5001 is a novel dual microtubule polymerization and PARP1/2 inhibitor [21]. AMXI-5001 showed an inhibitory effect comparable with that of clinical PARP inhibitors and polymerization inhibitors, and was assessed in a phase I/II trial in 2020 (NCT04503265). Ixabepilone (BMS-247550) is an analog of epothilone B, an orally bioavailable microtubule inhibitor, that also induces cell arrest at the G2-M phase of the cell cycle and subsequent apoptotic cell death in MDA-MB-468 (468) cells. A phase III trial in locally advanced or metastatic TNBC showed a longer progression-free survival (PFS, 4.2 months vs. 1.7 months) and a double objective response rate (RR) (31% vs. 15%) when ixabepilone was added to capecitabine compared to capecitabine alone [22].

### Targeting deficiency in homologous recombination

TNBC tumors are commonly linked with pathogenic mutations of BRCA1 and BRCA2. In total, 7–20% of patients with TNBC have BRCA1 or BRCA2 hereditary variants, and approximately 80% of BRCA1 mutations are detected in TNBCs [23]. BRCA1/2 mutations typically cause homologous recombination deficiency (HRD); thus, these tumors are susceptible to DNA crosslink agents or PARP inhibitor therapy. Table 2 presents phase III trials targeting homologous recombination in TNBC patients.

**Table 2:** Unpublished phase III trials targeting deficiency in homologous recombination

Drugs	Intervention	Register ID	Study population	Phase	Status
Olaparib	Olaparib to platinum-based neoadjuvant chemotherapy	NCT03150576	TNBC and/or germline BRCA BC	II/III	Recruiting
	Olaparib plus pembrolizumab versus chemotherapy plus pembrolizumab after induction with first-line chemotherapy plus pembrolizumab	NCT04191135	Locally recurrent inoperable or metastatic TNBC	II/III	Recruiting
Carboplatin	Paclitaxel and carboplatin with Olaparib	NCT03150576	TNBC and/or germline BRCA BC	II/III	Recruiting
	Doxorubicin, cyclophosphamide, paclitaxel, and carboplatin; or doxorubicin, cyclophosphamide, and paclitaxel	NCT02488967	Node-positive or high-risk node-negative TNBC	III	Recruiting
	Epirubicin and cyclophosphamide followed by paclitaxel with paclitaxel plus carboplatin	NCT03876886	TNBC with homologous recombination repair deficiency	III	Recruiting
	Doxorubicin, cyclophosphamide, and taxane; or doxorubicin, cyclophosphamide, taxane, and carboplatin	NCT02441933	TNBC	III	Recruiting
	Epirubicin, anthracycline, and paclitaxel; or epirubicin, anthracycline, paclitaxel, and carboplatin	NCT04296175	High-risk TNBC	III	Recruiting
	Weekly paclitaxel; or weekly paclitaxel and carboplatin	NCT03168880	Large operable or locally advanced TNBC	III	Active
	Carboplatin	NCT01752686	TNBC with pathologic residual cancer after neoadjuvant chemotherapy	III	Unknown

BC: breast cancer; BRCA: breast cancer susceptibility gene; and TNBC: triple-negative breast cancer

#### Platinum-based chemotherapy

Platinum salts can induce DNA crosslinking events subsequently leading to cell death. A phase II clinical trial (NCT00483223) with platinum monotherapy for mTNBC found that the patient RR was 25.6% in the overall population. For those with germline BRCA1/2 mutations, the RR was increased to 54.5% (95% confidence interval, CI, 23.4 to 83.3%), signifying that a proportion of germline BRCA1/2 mutations benefits most from platinum and that examination of tumor DNA repair function is necessary. This study also found that cisplatin was more active with an RR of 32.6% compared with 18.7% for carboplatin [23].

Currently, some cases without BRCA mutation exhibit biological features similar to those of BRCA-associated TNBCs called BRCAness, including BRCA1 mRNA-low, BRCA1 methylation, and HRD mutational signatures. In another phase III trial, unselected advanced BRCA1/2 mutated and BRCAness TNBC subgroups were treated with a platinum agent or docetaxel [24]. No significant differences in RR (31.4% vs. 34.0%), mean PFS (3.1 m vs. 4.4 m), or median OS (12.8 m vs. 12.0 m) were noted between carboplatin and docetaxel among all patients. The objective RR showed evidence of superiority with carboplatin (68.0%, 17/25) compared with docetaxel (33.3%, 6/18) in a BRCA1/2 germline mutation subgroup (*p* = 0.01). Similarly, a significantly longer PFS (6.8 m vs. 4.4 m) was also observed with carboplatin. However, patients with BRCA1 methylation did not benefit from carboplatin (21.4%) compared with docetaxel (42.1%, *p* = 0.28). The same result was observed in BC patients excluding those with BRCA1/2 mutation, indicating that not all BRCAness patients were suitable for platinum treatment. PrECOG 0105 focused on the genomic instability of BRCA1/2 mutation-associated breast cancer and TNBC in a phase II trial [25]. After neoadjuvant therapy consisting of carboplatin plus gemcitabine and iniparib (not a PARP1 inhibitor but involved in producing reactive oxygen), 36.3% (29/80) of patients achieved a pCR. The pCR rate was highest in TNBC patients with BRCA1/2 mutation (56%) followed by BRCA1/2 BC carriers (47%), and the lowest rate was noted in wild-type populations (33%). These findings support the view that identifying targeted populations is suitable for developing therapeutic strategies for HRD tumors.

The application of a platinum regimen in neoadjuvant therapy has been demonstrated to be an effective chemotherapy choice for patients beyond germline BRCA-mutated TNBC at the same time. In a phase II trial (GeparSixto; GBG 66, NCT01426880), nonmetastatic TNBC patients received carboplatin or no carboplatin with basic neoadjuvant paclitaxel and doxorubicin and additional bevacizumab [26]. A total 53.2% (84/158) of patients with carboplatin and 36.9% (58/157) of patients without carboplatin experienced a pCR (*p* = 0.005). In a secondary analysis of the GeparSixto trial, TNBC patients without germline BRCA1/2 mutations benefited from the addition of carboplatin. An increased pCR rate of 55% (66/120) was observed for patients with carboplatin compared with 36.4% (44/121) for patients without (*p* = 0.004) [27]. A similar benefit from carboplatin was observed in a phase III study (NCT01216111) among 647 operable TNBCs, comparing paclitaxel plus carboplatin (PCb) and cyclophosphamide, epirubicin, and fluorouracil followed by docetaxel (CEF-T). Increased 5-year DFS (86.5% vs. 80.3%, stratified log-rank *p* = 0.03), 5-year relapse-free survival (91.2% vs. 84.4%, p = 0.01), and distant DFS (92.6% vs. 87.9%, *p* = 0.05) were noted in the PCb group compared with in the CEF-T group [28]. For patients with stages II to III TNBC, a phase II trial (NCT00861705) aimed to evaluate carboplatin and/or bevacizumab on pCR after neoadjuvant therapy (paclitaxel followed by doxorubicin and cyclophosphamide) [29]. A total of 443 patients were randomly assigned into four arms to receive carboplatin and/or bevacizumab. According to the results, either carboplatin or bevacizumab significantly increased the pCR in the breast, whereas both agents achieved a 67% pCR breast rate. Approximately 60% (*n* = 221) of patients with carboplatin and 46% (*n* = 212) of patients without carboplatin achieved pCR (odds ratio = 1.76); simultaneously, 59% of patients in the bevacizumab treatment group achieved pCR compared with 48% (odds ratio = 1.58) of patients with TNBC who did not receive bevacizumab. Carboplatin alone significantly improved the pCR in the breast/axilla from 41 to 54%. Lobaplatin, another platinum agent, was tested in the clinical stages I to III TNBC ChiCTR-TRC-14005019 trial [30]. The addition of lobaplatin to NAC (docetaxel plus epirubicin) increased pCR in the breast and axilla as well as the overall RR. A significantly higher pCR rate (38.7% vs. 12.7%, odds ratio = 4.342, *p* = 0.001) and a better ORR (93.5% vs. 73.0%, odds ratio = 5.359, *p* = 0.003) were obtained in patients treated with the lobaplatin regimen. The hazard ratios of recurrence and metastasis were lower (*p* = 0.028) than those of docetaxel plus epirubicin alone in the follow-up. For histology-confirmed mTNBC, a phase III trial (NCT01287624) compared PFS with cisplatin or paclitaxel plus gemcitabine [31]. The median PFS (7.73 m vs. 6.47 m, hazard ratio of 0.692, *p* = 0.009) was superior for cisplatin plus gemcitabine compared to paclitaxel plus gemcitabine after follow-ups of 16.3 m and 15.9 m, respectively. Those studies have validated that the inclusion of platinum agents for early-stage TNBC and BRCA-mutated mTNBC could benefit long-term outcomes.

#### PARP inhibitors

The PARP cluster of polymerase enzymes controls genetic stability and DNA repair via the base excision repair pathway. Inhibition of PARP contributes to BRCA-mutated tumor cell death due to synthetic lethality. Currently, PARPi (e.g., olaparib, talazoparib, and veliparib) have been extensively applied in multiple cancers; however, those agents were not FDA approved for the treatment of locally advanced or metastatic breast cancer (mBC) until 2018.

The first PARPi assessed in a clinical study was olaparib, and its efficacy in BRCA1/BRCA2-mutated advanced BC was reported in 2010 [32]. Niraparib, veliparib, and talazoparib were subsequently developed and tested in different phases of clinical trials. Currently, talazoparib, which is derived from a by-product, is known as the most potent PARPi. Twenty operable HER2^−^ BC patients (15 TNBC) with germline BRCA positivity (16 germline BRCA1-positive and 4 germline BRCA2-positive patients) received talazoparib for six months and underwent a definitive surgical excision [33]. Residual cancer burden (RCB) was the primary endpoint. In this research, 53% (10/19) achieved RCB-0, indicating pCR, and 63% of patients were assessed as RCB-0/I. Among the different germline BRCA types, 53% of BRCA1-positive patients achieved RCB-0/I, and 100% (4/4) of BRCA2-positive patients had RCB-0/I. Subgroup analysis also showed that a higher RCB-0/I percentage was obtained in earlier stages (83% in T1, 54% in T2). These limited cases indicate that early PARPi intake may provide better benefits. The main toxic and adverse effects are associated with the hematologic system and are manageable by delayed delivery or supportive treatments. Four larger-scale multicenter phase II trials on talazoparib (NCT02401347, NCT04690855, NCT04755868, and NCT03901469) are ongoing.

Despite the promising efficacy of PARPi in TNBC, partial PARP-insensitive BRCA mutations of TNBC and acquired therapeutic resistance have been problematic in long-term studies [2]. Therefore, PARPi combined with platinum or other homologous recombination disrupting strategies for breast cancers have been researched to sensitize cancer cells. A phase II trial (NCT01042379) has been performed to evaluate veliparib plus carboplatin in HER2^−^ stage II or III breast cancer patients [34]. The percentage of patients who achieved a pCR was higher in the veliparib–carboplatin group (n = 72) than in control patients receiving standard neoadjuvant therapy (n = 44; 33% vs. 22%). Further analysis TNBC patients showed that pCR rates were 51% and 26% in the veliparib–carboplatin group and the control group, respectively. Nevertheless, studies on the addition of veliparib to carboplatin and standard chemotherapy in stage IIb–IIIc breast cancer and TNBC (NCT01818063) and the addition of veliparib to cisplatin (NCT02595905) in BRCA mutation-associated BC and/or mTNBC have been completed without published data. In 2018, a related phase III trial of veliparib–carboplatin in TNBC (NCT02032277) was reported [35]. In addition to the fundamental paclitaxel agent, carboplatin significantly improved the outcomes in pCR, RCB-0/I, a clinical breast tumor response, and eligibility for breast-conservation surgery. However, the addition of veliparib to carboplatin and paclitaxel did not significantly improve the outcome. In addition to the PARPi mentioned above, a study on rucaparib (NCT01074970) was completed in TNBC or ER/PR^+^, HER2^−^ patients with BRCA1/2 mutations.

PARP inhibitors in combination with immunotherapy have been further explored and apparently demonstrated superior antitumor activity. Niraparib plus pembrolizumab showed a 47% (7/15) objective RR, 80% (12/15) disease control rate, and 8.3 months of median PFS in patients with BRCA-mutated advanced or metastatic TNBC [36]. Several combinational therapies with small-molecule inhibitors, such as tyrosine kinase inhibitors (TKIs), have been evaluated in ongoing clinical trials given that preclinical studies have shown that inhibiting the PI3K pathway is likely to increase the response to PARPi. Olaparib and alpelisib (an inhibitor of α-specific PI3K) were evaluated in a dose-escalation and dose-expansion phase Ib trial among patients with epithelial ovarian cancer and breast cancer (NCT01623349). The results showed that 50% (14/28) of patients achieved stable disease and 36% (10/28) had a partial response according to response evaluation criteria [37]. Cediranib, an anti-angiogenic agent against vascular endothelial growth factor receptor (VEGFR) 1–3, was assessed with olaparib in a phase I trial (NCT03330847). Moreover, the combination of PARPi with an inhibitor of ATR serine/threonine kinase (ceralasertib, NCT03330847), a bromodomain and extra-terminal domain family of protein inhibitors (ZEN003694, NCT03901469), and an inhibitor of WEE1 G2 checkpoint kinase (adavosertib, NCT03330847) were evaluated under phase II studies. Table 2 presents unpublished phase III trials targeting deficiency in homologous recombination for TNBC patients.

### Androgen receptor targeted therapy

According to microarray analysis of TNBC molecular subtypes, the prognosis of LAR is related to decreased disease-free survival and poor overall survival. However, LAR subtype expresses androgen receptor, and its growth is driven by androgen signaling [38]. Meta-analyses noted that 27.96% (1315/4703) of TNBC patients expressed AR [39]. In a phase II study (NCT01889238) that evaluated the expression of nuclear AR to screen eligible population candidates for AR inhibition, approximately 80% (n = 368) of patients with TNBC expressed AR in the nucleus, and approximately 55% of patients expressed AR greater than 10% of cells [38]. In this trial, enzalutamide, an AR antagonist that potently plays multiple roles in the AR signaling pathway, was tested in advanced AR-positive (nuclear AR ≥ 10%) patients with TNBC. The 118 AR-positive patients were treated in the intent-to-treat population, and 78 patients were treated in the evaluable subgroup. Clinical benefit rates of 33% and 28% were observed at 16 weeks and 24 weeks, respectively, and 8% achieved CR or PR in the evaluable subgroup. The secondary endpoints, including median PFS and OS, were 3.3 m and 17.6 m, respectively, suggesting that enzalutamide is effective in the treatment of advanced AR^+^ TNBC.

Gucalp and colleagues reported the results of bicalutamide treatment for 424 patients with AR^+^ER^−^PR^−^ mBC, and 12% of these were AR > 10%. Finally, 26 cases completed the study regimen with a clinical benefit rate of 19% at 6 months and a median PFS of 12 weeks [40]. In addition, some promising AR inhibitors are currently in preclinical studies that are also expected to be introduced to TNBCs. For example, ZETA55, a novel dual AR and histone deacetylase (HDAC) 6 inhibitor, is a promising therapeutic agent that selectively inhibits HDAC6 activity, leading to AR degradation and preventing its nuclear translocation [41].

Abiraterone acetate irreversibly inhibits cytochrome P450 family 17 subfamily A, polypeptide 1 (CYP17A1, a rate-limiting enzyme in androgen synthesis) enzymatic activity and is widely prescribed for resection-resistant prostate cancer. Abiraterone acetate plus prednisone was assessed in 30 patients with AR^+^ advanced mTNBC. This trial observed that the clinical benefit rate (CBR) at 6 m was 20.0%, the median PFS was 2.8 m, and the objective RR was 6.7% [42]. Other drugs, such as inhibitors of cytochrome p450 family 17 (VT-464, orteronel) and dehydroepiandrosterone, are being assessed in clinical trials, and the results are eagerly awaited.

Enobosarm (GTx-024), a nonsteroidal selective androgen receptor modulator, has been shown to be effective in AR^+^ BC. Recently, enobosarm in combination with pembrolizumab was reported to have good tolerability in 16 cases with AR^+^ mTNBC (NCT02971761) [43]. The results revealed that 4 of 16 (25%) patients achieved a CBR at 16 weeks with a PFS of 2.6 m and an OS of 25.5 m. Unfortunately, the study was terminated prematurely due to the GTx-024 drug supply. In future trials, the combination of antiandrogen-related targeted therapy with immune checkpoint blockade (ICB) for AR^+^ TNBC is worthy of attention. In addition to AR-positive TNBC subtypes, partial LAR subtype cell lines have a high frequency of PIK3CA mutations with AR dependency, resembling ER-positive breast cancers. In addition, a synergistic effect of combining bicalutamide with a PI3K inhibitor was observed in preclinical data [44]. Related AR-positive TNBC clinical trials are shown in Table 3. To conclude, AR targeted therapy has high potential to treat AR-positive TNBC subtypes.

**Table 3:** Ongoing clinical trials aimed at androgen receptor-positive TNBC

Drugs	Pharmacological mechanism	Register ID	Phase	Status
Bicalutamide	AR antagonists	NCT02348281	II	Terminated
		NCT03055312	III	Terminated
		NCT02353988	II	Unknown
Enzalutamide	AR pathway inhibitors	NCT02689427	IIb	Recruiting
		NCT02750358	II	Active
Seviteronel	A potent CYP17 lyase inhibitor	NCT02130700	II	Completed
		NCT02580448	I/II	Completed
MK-2866	A nonsteroidal selective AR modulator	NCT02368691	I	Terminated
Darolutamide	Competitively inhibiting AR binding, translocation, and transcription	NCT03383679	II	Recruiting
Dehydroepiandrosterone	Intermediates of steroid hormones	NCT00972023	I	Terminated
Orteronel	CYP17 inhibitors	NCT01990209	II	Active

AR: androgen receptor; CYP17: 17-[α]-hydroxylase/17:20-lyase (CYP17)

### PI3K/AKT/mTOR pathway inhibition

The PI3K/AKT/mTOR signaling pathway is frequently activated in processes involved in tumorigenesis, cancer cell proliferation, survival, and resistance to anticancer therapies. The pathway also plays a crucial role in TNBC as mutations and activation in PI3K or AKT1 and loss of PTEN are often noted in TNBC. The frequency of genomic alteration in PI3K is second after TP53 in TNBC, and interestingly, it is significantly rarer (~ 10%) [45] in TNBC compared to other breast cancer subtypes (34.5% in HR^+^ BC and 22.7% in HER2^+^ BC) [46]. The incidence rate of PIK3CA mutation is more common in residual TNBC and AR-positive TNBC, whereas amplification of AKT3 and deletion of PTEN are elevated in the basal subtype. Patients with PIK3CA-mutated TNBC have a longer median OS after targeted treatment than those with PIK3CA wild-type TNBC (NCT02299999) [47]. Inhibitors of this pathway, such as ipatasertib (an AKT inhibitor), buparlisib (a PI3K inhibitor), everolimus (an mTOR inhibitor), and capivasertib (an AKT inhibitor), have been evaluated for their antitumor response in clinical trials.

#### PI3K inhibitors

Class I PI3K inhibitors, such as buparlisib (BKM120), have been shown to be effective in inducing TNBC tumor regression. The phase II clinical trial on buparlisib has been launched, showing a median OS of 11.2 m, a median PFS of 1.8 m, and a clinical benefit rate of 12% in 50 cases with mTNBC. Regarding the safety of treatment-related adverse events, the most common symptoms included fatigue (58%), hyperglycemia (34%), nausea (34%), and anorexia (30%) [48]. BELLE-4 is a phase II/III study (NCT01572727) for the treatment of advanced HER2^−^ BC patients with buparlisib combined with paclitaxel; however, no improvement in PFS either in the recruited population or in the activated PI3K population was achieved compared with placebo; in addition, the occurrence of adverse events could not be neglected [49]. Buparlisib plus LDE225 (vismodegib, a smoothened inhibitor) has been evaluated in NCT01576666, and the results have not been made public.

Previous studies verified sensitization to PARP inhibitors after PI3K inhibition in BRCA-proficient TNBC and TNBCs without BRCA mutations, providing a rational theoretical basis for combining PI3K and PARP inhibitors [50, 51]. A phase I clinical trial with buparlisib and olaparib was initiated and observed a 9/12 response in BC patients with germline BRCA mutation and a 3/5 response in patients with wild-type BRCA [52]. The application of alpelisib monotherapy or other PI3K inhibitors plus PARPi applied in TNBC still warrants further investigation.

#### AKT inhibitors

Ipatasertib, a potent small-molecule kinase inhibitor that is highly specific to AKT and competes for ATP, demonstrates efficacy in various cancer cells, e.g., ovarian, colorectal, non-small cell lung, and breast cancers. Early studies concluded that ipatasertib sensitivity was mainly related to high phosphorylated AKT levels, PIK3CA mutation, and PTEN mutation or deficiency, whereas resistance to ipatasertib tended to be associated with KRAS and BRAF mutations. In a phase II randomized placebo-controlled LOTUS trial [53], ipatasertib was additionally added to paclitaxel as first-line therapy in TNBC patients. These results preliminarily suggested that targeted AKT benefits patients with TNBCs given that the median PFS was prolonged in the ipatasertib group (6.2 m vs. 4.9 m, p = 0.037). Moreover, in the subgroup of PIK3CA/AKT/PTEN-altered tumors, the median PFS was 6.2 months with ipatasertib versus 3.7 months without ipatasertib (p = 0.041), revealing significantly improved survival outcomes. However, in the FAIRLANE trial, ipatasertib did not demonstrate a statistically significant increase in the pCR rate in early TNBC [54].

Capivasertib is another highly selective small-molecule inhibitor targeting AKT1-3. Similar to ipatasertib, the sensitivity to capivasertib mainly depends on PI3K/AKT activation and/or PTEN status. The preclinical antitumor activity of capivasertib was first tested in animal models; furthermore, the PAKT trial (NCT03997123) evaluated the efficacy and safety of the addition of capivasertib to paclitaxel among TNBC patients [55]. After a median follow-up of 18.2 months, the median PFS, OS, and duration of response tended to be longer with capivasertib compared with placebo in the intent-to-treat group. In the intent-to-treat group, 28 patients with PIK3CA/AKT mutations or PTEN alterations comprised 25% of the total analyzable samples. In this genetically abnormal subgroup, capivasertib significantly enhanced the benefits in PFS and duration of response: 9.3 months of median PFS with paclitaxel plus capivasertib and 3.7 months with paclitaxel plus placebo; 13.3 months of median duration of response with capivasertib and 3.5 months with placebo. Regarding the safety of capivasertib, the most common grade ≥ 3 adverse events were acceptable: diarrhea, infection, neutropenia, rash, and fatigue. Both phase II (NCT02423603) and phase III (NCT03997123) studies of the addition of capivasertib to paclitaxel have been registered on the clinical trials website.

#### mTOR inhibitor

The mTOR inhibitor, everolimus, has been applied in combination with lapatinib (NCT01272141), cisplatin (NCT01931163), and carboplatin (NCT02531932) in TNBC-related trials. An open-label phase II clinical trial aimed to assess RCB with everolimus plus cisplatin treatment among 24 stage II/III TNBC patients after NAC [56]. In this trial, 22 cases were enrolled in the efficacy analysis and 5 cases achieved RCB-0/I at surgery with an RR of 23%. These RCB-I patients were analyzed by somatic mutation testing and germline mutation testing, revealing actionable somatic PIK3CA mutations in 2 cases and germline partner and localizer of BRCA2 (PALB2) mutation in 2 cases. Given limited enrollment in this trial, further investigation of the relationship between efficacy and PI3KCA mutations is needed to optimize the treatment regimen.

The PI3K pathway is not only involved in drug resistance; inhibitors, including PI3K inhibitors, are also prone to intrinsic tolerance. Some progress has been made regarding the issue of drug resistance in TNBC patients. Juric et al. found that resistance to PI3K/AKT inhibitors mainly relied on PTEN deficiency, which accounts for 35% of TNBCs [57, 58]. Histone demethylase lysine demethylase 4B (KDM4B) represents an important target, leading to preferential apoptosis in PTEN-altered TNBC. Moreover, synergistic effects are noted when combined with the PI3K inhibitor pictilisib [59]. The frequently activated Notch pathway in breast cancers has also been involved in resistance to PI3K inhibitors [60]. Residual mTORC1 activity was sustained with the 3-phosphoinositide-dependent kinase 1-serum/glucocorticoid-regulated kinase 1 axis, and suppression of either the 3-phosphoinositide-dependent kinase 1 or serum/glucocorticoid-regulated kinase 1 could restore sensitivity to PI3K inhibition in resistant cells [61]. Therefore, the combination of drugs is a principal method to avoid drug resistance [59].

### Antitumor angiogenesis agents

#### Bevacizumab

Vascular endothelial growth factor and its tyrosine kinase receptor VEGFR play an important role in the invasiveness of a variety of solid tumors. Anti-angiogenic therapies, i.e., the monoclonal antibody bevacizumab and several small-molecule tyrosine kinase inhibitors, such as apatinib have become available and have generated dramatic therapeutic responses [62]. However, targeted therapy with VEGF and VEGFR has yielded contradictory results in breast cancer. Bevacizumab benefited patients with breast cancer in early research and was rapidly approved by the FDA, but the subsequent data did not support its ability to boost overall survival or quality of life [63]. The combination of bevacizumab with chemotherapy was demonstrated to improve pCR in stage II to III TNBC, as mentioned before [29]. Bevacizumab has also been shown to improve pCR (39.3% vs. 27.9%, *p* = 0.003) when added to NAC treatment in patients with TNBC [63]. In the GeparQuinto phase III trial (NCT00567554), TNBC patients were treated with anthracycline and taxane, and the addition of bevacizumab yielded a higher pCR rate in BRCA1/2 mutation carriers (61.5% vs. 35.6% in the nonmutated group, *p* = 0.004). However, the overall pCR rate in the BRCA1/2 alteration subgroup was essentially improved compared with those without mutations (OR, 2.17; p = 0.001) [64]. No statistically significant benefit was found in CALGB 40,603, and the lack of long-term survival rate data failed to support its use in combination with bevacizumab. In addition, the risk–benefit ratio was also questioned due to clearly increased toxicity after the addition of bevacizumab [29]. In general, bevacizumab still rarely meets patient expectations.

#### VEGFR inhibitors

VEGFR kinases such as apatinib and lenvatinib represent substitutes for bevacizumab. Apatinib is a novel highly selective antitumor agent that blocks VEGFR2 signaling. It was found that pVEGFR2 is a biomarker of populations sensitive to anti-VEGF agents based on Cox and logistic regression models in 80 apatinib-pretreated advanced BC patients [65]. Forty patients with advanced TNBC were enrolled in a phase II clinical trial. The patients were treated with camrelizumab as well as continuous dosing or intermittent dosing of apatinib to evaluate the disease control rate and PFS [66]. The continuous dosing cohort had a higher disease control rate (63.3% vs. 40.0%) and longer PFS (3.7 m vs. 1.9 m) than the apatinib intermittent dosing cohort, supporting the use of camrelizumab combined with apatinib in patients with advanced TNBC. ENMD-2076 is an aurora-A kinase inhibitor with anti-angiogenic properties that has shown activity in preventing proliferation and promoting apoptosis in preclinical models of TNBC. A single-arm, two-stage phase II trial aimed to treat ENMD-2076 until unacceptable toxicity or disease progression occurred in patients with previously treated advanced TNBC or mTNBC [67]. A total of 2/41 of patients exhibited partial responses, and 16.7% of patients achieved a 6-month clinical benefit rate, demonstrating favorable therapeutic effects.

### Epigenetic modifications inhibiting

Epigenetic modifications, including DNA modifications (such as DNA methylation) and histone modifications (such as histone deacetylation and lactylation), often regulate gene expression, which may represent a promising therapeutic strategy to make hormone negative TNBC susceptible to endocrine therapy. Laboratory studies have demonstrated that ER was present in some TNBCs, but it was “silenced” due to inactivation by methyl and histone groups. Particular drugs called demethylating inhibitors (such as decitabine) and HDAC inhibitors can remove these methyl and histone groups and reactivate the ER, thus providing an opportunity for epigenetic therapy and reintroduction of endocrine therapy for TNBC [68]. Decitabine (5-aza-2′-deoxycytidine) is an FDA-approved DNA methyltransferase inhibitor that has demonstrated antitumor activity in hematological neoplasms. The application of decitabine to treat breast cancer has revealed an acquired response in patients, but the main limitation is due to the small sample size. NCT04722978 is a phase III study planned to treat mTNBC with moxifloxacin, gemcitabine, and carboplatin. A study (NCT01105312) focused on panobinostat (LBH589, an HDAC inhibitor) combined with letrozole and published its safety and recommended dose in mBC [69]. However, given the limited number of cases, especially those with measurable disease, the therapeutic response of the combination lacks strong evidence. CUDC-907 (a dual-action inhibitor of HDAC1/2/3/10 and PI3Kα, NCT02307240) is another HDAC inhibitor studied in clinical trials as a monotherapy. Other combination therapy trials containing HDAC inhibitors include NCT04315233 (belinostat in combination with ribociclib), NCT04192903 (chidamide plus cisplatin), NCT02393794 (romidepsin with nivolumab), and NCT02708680 (entinostat in combination with atezolizumab).

In addition to being epigenetically involved in gene repression and phenotype features, CDK2 also modulates phosphorylation of the zeste homolog 2 enhancer to maintain the TNBC phenotype. Inhibition of CDK2 transforms TNBC into the luminal ERα-positive subtype and makes it sensitive to tamoxifen [70]. The combination of a CDK2 inhibitor or zeste homolog 2 enhancer inhibitor with tamoxifen markedly suppresses tumor growth and effectively improves the outcomes of mice bearing TNBC tumors. Zhang et al. identified a new ER-α36, which was named based on its molecular weight at of 36 kDa, and this protein differs from the commonly studied ER-α66. ER-α36 was discovered in both ER-positive breast cancer cells and TNBC cell lines, and a subsequent study in TNBC identified a positive feedback loop of ER-α36/EGFR, indicating responsiveness to mitogenic estrogen signaling in ER-α36 expressing TNBC [71, 72]. Roswall et al. demonstrated that inhibition of the paracrine platelet-derived growth factor-CC signaling between stromal and cancer cells in the microenvironment could enhance the previously resistant basal-like, hormone receptor-negative BC subtype, which is more sensitive to endocrine therapy [73].

### Other antitumor targets

#### EGFR

Epidermal growth factor receptor (EGFR) is overexpressed in approximately 70–78% of basal-like TNBC samples [74], thus providing a therapeutic target for EGFR-related targeted therapy, although TNBC is characterized by a lack of HER2. A preclinical investigation demonstrated that afatinib has good antitumor activity in 14 TNBC cell lines with IC_50_ values ranging from 0.008 to 5.0 µM, especially in the basal-like subtype of TNBC [75]. Combining afatinib with other targeted drugs could enhance growth inhibition. Mechanistically, afatinib exerts its antiproliferative effects on dasatinib by arresting the G1 cell cycle and inhibiting both pERK (T202/T204) and pAkt (S473) signaling. EGFR-based nimotuzumab (NCT01939054), panitumumab (NCT02876107, NCT02593175), and SCT200 (NCT03692689) have already been evaluated in clinical studies. Inhibitors such as dasatinib (BMS-354825) and gefitinib have been tested in advanced TNBC. In a phase II study (NCT00371254), the objective RR for dasatinib with advanced TNBC was 4.7%, demonstrating the limited therapeutic activity of single-agent dasatinib [76]. An early phase II clinical study showed no complete or partial response in 31 patients with advanced BC treated with gefitinib monotherapy [77].

In addition, EGFR-targeted nanoparticles with paclitaxel and cetuximab enhanced mitotic catastrophe and apoptosis, providing a feasible strategy for TNBC therapy [78]. Liposomal targets of EGFR (anti-EGFR-IL-dox, NCT02833766) and ADCs to human epidermal growth factor receptor 3 (U3-1402, NCT04699630) are being tested in patients with advanced BC. Unfortunately, these trials are under recruitment or have been terminated, and data have not been published.

#### FGFR

Activation of fibroblast growth factor receptor (FGFR) is common in many tumor types, and a good response to FGFR inhibition might be beneficial. FGFR is amplified in greater than 10% of breast cancers and approximately 4% of TNBC. However, FGFR is thought to regulate the development of TNBC. Higher-level clonal amplification of FGFR, especially FGFR2, has a higher RR to selective FGFR inhibitors [79, 80]. In this translational clinical trial, 8 breast cancer patients had FGFR1 amplification and were treated with an FGFR inhibitor (AZD4547), and a respond was confirmed in one patient. Erdafitinib, the first approved FGFR-targeted agent in urothelial carcinoma, followed by the FGFR-targeted selective inhibitors dovitinib and lucitanib were also applied in FGFR pathway-amplified breast cancer [81]. FGFR-TKI resistance caused by gatekeeper residue mutations has raised concern in other cancers [82]. Therefore, novel FGFR inhibitors, such as LY2874455 [83] and 7v [84], do not target gatekeeper residues, and multitarget kinase inhibitors, such as ponatinib [85], have been developed to overcome mutation-based FGFR-TKI resistance. However, anti-FGFR target treatment requires strict long-term assessment processes before being widely used in TNBC.

#### CXCR4

C-X-C chemokine receptor type 4 (CXCR4) is overexpressed in over 20 cancer types, including breast cancer, and is a key mediator of cancer intracellular signaling pathways and cell trafficking, correlating with aggressive phenotypes and poor prognosis. The novel CXCR4 antagonist balixafortide (POL6326, NCT01837095) was assessed in combination with eribulin in patients with pretreated, relapsed metastatic HER2-negative BC [86]. The preliminary antitumor activity seems promising in evaluated patients. A clinical benefit was observed in 44% (24/54) of patients, and the objective RR was 30% (16/54). In the dose-escalation assessment, the tolerability and safety of balixafortide plus eribulin were acceptable and similar to monotherapy with eribulin or balixafortide. In addition, the development of a receptor-based peptide, a CXCR4-binding peptide (DV1), has been shown to be associated with reduced cell migration and inhibited metastasis in vitro and in preclinical models [87]. CXCR4 also mediates sensitivity to endocrine and anti-PDL-1 therapy in vitro [88]; thus, inhibiting CXCR4 with endocrine therapy and immune checkpoint therapy represents the direction of future exploration.

#### TP53-related rescue

The human tumor suppressor gene TP53, which is frequently mutated or inactivated in approximately 60% of cancers, was also found to be the most common alteration in both post-NAC TNBC (72–89%) and TCGA (~ 85%) [14, 89]. Therefore, TP53-targeted therapy is currently under development, especially inhibitors with a synthetic lethality effect. Compounds such as PRMIA-1 and APR-246 were previously reported to rescue mutant TP53 TNBC cells by inhibiting cell proliferation and migration and inducing apoptosis and exhibited synergistic therapeutic effects with olaparib [90]. The antitumor activity and safety of AZD1775, an inhibitor of WEE1, were assessed in a phase Ib study of patients with advanced solid tumors (NCT02482311).

#### Emerging metabolism-related strategies

Other precise treatments have not yet been applied in the clinic but have been shown to be promising in basic research, such as inhibition of the unique reactivated pathway, metabolic biosynthesis pathway, endocrine therapy after induction, epigenetic treatment, and autophagy initiator. Metabolic disturbance is one of the top ten hallmarks of tumors. Excessive accumulation of cholesterol esters and metabolites in cancer cells promotes malignant activity, such as proliferation, metastasis, and therapeutic resistance [91]. Interest in fatostatin, a small molecule that targets sterol regulatory element-binding proteins, has waned given its toxicity. The cholesterol-biosynthesis pathway is also correlated with the responses and activities of tumor microenvironments; thus, developing novel low-toxicity inhibitors of the metabolic pathway would provide a new therapeutic strategy. Several novel targeted small-molecule drugs have been discovered to induce cancer cell death, such as the autophagy initiator LYN-1604 (unc-51-like autophagy activating kinase 1 activator) [92], smoothened inhibitor NVP-LDE225 (sonidegib) [85], myeloid cell leukemia-1 inhibitor S63845 [93], and antioxidative stress oral gold-containing drug (auranofin) [94]. These drugs exerted antitumor activity in vitro either alone or in combination with currently used chemotherapy agents. These agents are likely to be used in therapeutic strategies for TNBC and are worthy of further assessment in preclinical and clinical studies.

#### Cancer stem cells-related strategies

Cancer stem cells (CSCs), which are also known as tumor-initiating cells, have the ability to self-renew to drive the process of tumorgenesis and differentiation, contributing to cancer cells heterogeneity [95]. Compared to nonstem tumor cells, BC CSCs proliferated more slowly and exhibited a higher degree of chemoresistance [96]. Evidence from multiple studies suggests that the TGF-β pathway is involved in the maintenance of BC CSCs [97–99]. TNBC chemoresistance is highly correlated with CSCs. Chemotherapy-induced TGF-β signaling promotes tumor recurrence via IL-8-dependent CSC expansion, and inhibition of TGF-β stops the development of drug-resistant CSCs [100]. Furthermore, the presence of TGF-β in the breast tumor microenvironment induced angiopoietin-like 4 expression through the Smad pathway and initiated metastasis of cancer cells to the lung [101]. All of these findings suggest that there is an opportunity for TGF-β pathway intervention in TNBC. A clinical trial is currently investigating the side effects and optimal dose of galunisertib (TGF-β inhibitor) in combination with paclitaxel in the treatment of patients with metastatic AR-negative TNBC (NCT02672475). Bintrafusp alfa (M7824) is a bifunctional fusion protein targeting TGF-β and PD-L1 that is currently being evaluated. A phase Ib trial (NCT03579472) is assessing the side effects and optimal dose of bintrafusp alfa in combination with eribulin mesylate for the treatment of metastatic TNBC, and a phase II trial (NCT04489940) is testing its efficacy as a monotherapy in TNBC patients.

In addition, different studies have shown that the Notch [102], Wnt [103], and JAK/STAT [104] pathways are involved in the maintenance of BC CSCs, and the corresponding inhibitors are under clinical investigation. A phase II trial (NCT04461600) is currently investigating the efficacy and safety of the Notch inhibitor AL101 as monotherapy, whereas clinical trials of two other inhibitors, PF-03084014 (NCT02299635) and RO4929097 (NCT01151449), have been terminated. LGK974, a Wnt-specific acyltransferase, effectively inhibits the Wnt/β-catenin pathway, and a phase I trial (NCT01351103) is currently assessing its recommended dose in TNBC patients. Based on the results of ruxolitinib, a JAK1/2 inhibitor that has shown good tolerability in combination with paclitaxel for HER2-negative BC treatment [105], a phase II trial (NCT02876302) of ruxolitinib plus paclitaxel for TNBC is underway. TTI-101 is a competitive inhibitor of the STAT3 pathway and has been shown to have powerful antitumor activity in preclinical studies in several cancer models, including non-small cell lung cancer [106], head and neck squamous cell carcinoma [107], hepatocellular carcinoma [108], and palbociclib-resistant ER-positive BC [109]. A phase I trial of TTI-101 in advanced tumors, including TNBC, is currently recruiting patients (NCT03195699).

#### Other important pathways

Inhibitors of apoptosis proteins (IAPs) are the key negative regulators of programmed cell death. Those proteins are upregulated in most tumors to promote cancer cell survival and induce treatment resistance [110], and targeting IAPs is another promising approach for the treatment of TNBC. In 2000, Wang et al. identified second mitochondria-derived activator of caspases (SMAC) as an endogenous antagonist of IAPs [111], and the crystal structure of the SMAC/DIABLO complex was solved, providing the basis for the development of small-molecule antagonists of IAPs [112]. DEBIO1143, a small-molecule mimetic of SMAC [113], has been shown to inhibit the growth of multiple cancer cell lines in preclinical studies and to enhance the therapeutic effect of radiotherapy and chemotherapy in mouse models; however, DEBIO1143 has only been tested in phase I clinical trials in patients with advanced solid tumors [114]. Another SMAC mimetic, LCL161, entered a phase II trial (NCT01617668) in patients with TNBC. In this study, neoadjuvant treatment with LCL161 and paclitaxel showed promising signs of efficacy in TNBC patients with TNFα-based gene expression signature positivity [115].

Heat shock protein 90 (Hsp90) is a chaperone protein frequently expressed in breast cancer [116] that stabilizes the structural and functional integrity of many oncogenic clients and can act as a protective “biochemical buffer” [117]. Onalespib (AT13387) is an inhibitor that targets the N-terminal ATPase domain of HSP90 [118] and showed modest antitumor activity in phase I trials in patients with advanced solid tumors [119, 120]. Onalespib plus paclitaxel is currently being studied for the treatment of TNBC patients in a phase Ib trial (NCT02474173). In addition, SL-145, a novel C-terminal inhibitor of HSP90, has demonstrated antitumor and antimetastatic effects on TNBC cells in a preclinical study and may represent a promising agent in the future [121].

## Immunotherapy for TNBC

TNBC is suitable for immunotherapeutic treatments mainly due to tumor immune infiltration, neoantigens caused by mutational burden and higher genomic instability, and high levels of immune markers such as PD-L1 and programmed cell death protein-1 (PD-1), which are closely correlated with the tumor response, relapse, and overall outcomes. Immunotherapy has demonstrated efficacy in various neoplasms; thus, immunotherapeutic interventions against TNBC hold great promise. Among various types of immunologic options, molecular and cellular immunotherapies have exhibited significant potential based on evidence provided by preliminary study outcomes [122]. The FDA approved atezolizumab for programmed death-ligand 1 (PD-L1)-positive unresectable locally advanced or mTNBC on March 8, 2019, representing the earliest ICB monoclonal antibody (mAb) approved for TNBC. Later, pembrolizumab in combination with chemotherapy for locally recurrent unresectable or mTNBC patients with positive PD-L1 expression (CPS ≥ 10) was also approved by the FDA on November 13, 2020 [123].

The main factor involved in tumor cell immune infiltration in TNBC is tumor-infiltrating lymphocytes (TILs). TILs interact with tumor cells, modify the tumor immune microenvironment, and participate in Th1-cell immune response attack or immune suppression [124]. Sylvia Adams and colleagues evaluated the density of TILs in stromal (sTILs) and intraepithelial compartments (iTILs) from 506 TNBC tumor samples. In total, 80% of cancers had ≥ 10% sTILs (10–80%), but only 15% of tumors had ≥ 10% iTILs (10–50%). sTILs rather than iTILs were confirmed to be independent prognostic factors of good prognosis. A 14% reduction in recurrence or death risk, 18% reduction in distant recurrence risk, and 19% reduction in death risk were noted with a 10% increase in TILs after a median of 10.6 years of follow-up [125]. Therefore, therapeutic approaches that promote the infiltration of immune cells into tumor tissue as well as activation, such as chimeric antigen receptor T (CAR-T), hold significant promise. In addition to tumor stromal compartments, high expression levels of the classic immune checkpoint molecules, PD-L1 and PD-1, are well-established targets for immunotherapy in some solid tumors. PD-1/PD-L1 was reported to be commonly expressed in breast cancers, especially in TNBC. In a study with 53 cases of TNBC, up to 70% and 59% expression levels of PD-1 and PD-L1, respectively, were noted, whereas both PD-1 and PD-L1 were expressed in 45% of samples [126]. PD-L1 and PD-1 are important ICB molecules because PD-L1 from cancer cells can integrate with PD-1 on T cells, making it easier for cancer cells to avert T cell-mediated immune response. Thus, blocking their interaction with monoclonal antibodies will reactivate TILs, which has positive clinical effects in many tumors not limited to TNBC [122]. Demonstrating the highest mutational frequency among breast cancer subtypes, TNBC has significant genomic instability and potentially creates neoantigens discerned by the immune system. These features provide strong evidence that TNBC treatment is entering the era of immunotherapy.

### Immune checkpoint blockade with monotherapy

As noted on clinicaltrials.gov [19], approximately half of the registered studies are focused on immune checkpoint blocking-related therapies. Of these, greater than 100 clinical studies have already entered phase II or phase III, implying that immunotherapy is an important trend in TNBC treatment. Previous trials have shown positive results with pembrolizumab or atezolizumab monotherapy in TNBC. In the KEYNOTE-012 trial (NCT01848834), 27 PD-L1-positive TNBC patients exhibited an ORR of 18.5%, and the median time to response was 17.9 weeks [127]. Another targeting PD-L1 mAb, atezolizumab, was also reported to be safe and clinically active in mTNBC. In this phase I study (NCT01375842) [128], the evaluation of PD-L1 expression levels demonstrated an improved ORR, a longer OS, and a higher disease control rate in patients with at least 1% TILs expressing PD-L1. Interestingly, patients receiving first-line atezolizumab therapy exhibited a better prognosis (e.g., higher ORR, median OS compared to those receiving second-line or next), suggesting the superiority of atezolizumab combined with first-line.

### Combinations of immune checkpoint inhibitors

However, most patients with TNBC do not respond well to PD-1 or PD-L1 monotherapy; therefore, inducing a favorable tumor immune microenvironment appears to be particularly important. Conventional chemotherapeutic agents, such as taxane, cisplatin, and cyclophosphamide, can enhance tumor antigen release, improve the tumor microenvironment, and add the possibility of an antitumor response [129–131]. Biopsies before and after NAC showed that the immune microenvironment was altered from low TIL to high TIL, and patients with high TIL levels exhibited improved survival [132]. For example, paclitaxel has pleiotropic immune-modulating effects because it helps mature dendritic cells shift the T-helper phenotype to promote the secretion of proinflammatory cytokines and enhance the activity of CD8^+^ T cells [129]. An animal model has shown evidence that cisplatin markedly induces tumor regression and improves survival when combined with anti-PD-1 and anti-cytotoxic lymphocyte antigen 4 (CTLA4) [133]. These studies suggest that ICB combined with chemotherapy may achieve a synergistic or additive clinical effect.

The efficacy of atezolizumab plus nab-paclitaxel for locally advanced TNBC or mTNBC was assessed in the phase III Impassion130 trial (NCT02425891) [134]. In this report, the median PFS was 7.2 m with atezolizumab and 5.5 m without atezolizumab. Among patients with PD-L1^+^ (PD-L1 expression of infiltrated immune cells accounted for ≥ 1% of the tumor area), the median PFS was 7.5 m with atezolizumab compared to 5.0 m in those without atezolizumab. However, in Impassion131 (NCT03125902), atezolizumab with paclitaxel failed to improve PFS or OS compared with paclitaxel alone [135]. A new study (Impassion031, NCT03197935) on atezolizumab combined with chemotherapy in early-stage TNBC has shown significantly improved pCR rates. The pCR was 58% (95/165) in the atezolizumab group and 41% (69/168) in the placebo and chemotherapy groups. In the PD-L1-positive population, the pCR rates in the atezolizumab and placebo groups were 69% and 49%, respectively [136].

Pembrolizumab was approved based on results from the KEYNOTE-355 (NCT02819518)[137]. KEYNOTE-355 enrolled untreated advanced TNBC patients to receive pembrolizumab plus chemotherapy. The median PFS was 9.7 m (95% CI: 7.6–11.3) in the pembrolizumab arm and 5.6 m (95% CI: 5.3–7.5) in the placebo arm (p = 0.0012) among patients with CPS ≥ 10, indicating that PD-L1 enrichment affected pembrolizumab treatment. In the KEYNOTE-522 (NCT03036488) trial, pembrolizumab was added to NAC (paclitaxel and carboplatin-based) in patients with early TNBC. The pCR was significantly higher in the pembrolizumab group (64.8%) than in the placebo group (51.2%, p < 0.001); the percentage of disease progression that precluded definitive surgery was lower among those who received pembrolizumab (7.4%) than among those who received in the placebo group (11.8%) [138]. However, pembrolizumab did not improve median OS in previously treated mTNBC regardless of PD-L1 CPS results from KEYNOTE-119 (NCT02555657)[139]. These findings reveal that patients benefit more from early immunotherapy and underscore the need to screen appropriate subgroups for immunotherapy.

A preclinical study revealed crosstalk between PARPi and PD-L1 blockade. In cellular and animal models, PARPi alone mediated increased expression of PD-L1, whereas blocking PD-L1 resensitized cells to PARPi. These findings indicate that PARPi alone can reduce the anticancer effect through immune tolerance, but the combination of PARPi and PD-L1 blockade intensified the therapeutic efficacy [140]. TCGA dataset set also provided evidence that BRCA1-mutated tumors have higher levels of tumor-specific neoantigens, recruiting a prominent lymphocytic infiltrate and leading to a more robust higher T cell response than BRCA1-wild-type TNBCs [133], supporting a rational strategy for immunotherapy in combination with DNA repair targeted agents in BRCA1-associated TNBC. The results of pembrolizumab with niraparib in BRCA1-mutated TNBC have been described previously [36]. Another phase II/III study (NCT04191135) exploring the addition of olaparib to pembrolizumab plus chemotherapy in patients with advanced TNBC or mTNBC is almost complete. NCT03801369 and NCT03167619 are studies assessing durvalumab plus olaparib in advanced TNBC. NCT04690855 explored atezolizumab plus talazoparib and high-dose radiation in PD-L1-positive mTNBC.

In addition to the combination of PARPi and immunotherapy, a series of trials of immunotherapy in combination with other drugs have entered clinical studies (Table 4). For example, alternative PD-1/PD-L1 ICB combined with tyrosinase inhibitors has entered phase III studies (NCT04177108, NCT04335006, and NCT04405505).

**Table 4:** Unpublished phase III trials of immunotherapy for TNBC

Drugs	Intervention	Register ID	Study population	Status
Atezolizumab	Atezolizumab to carboplatin and nab-paclitaxel	NCT02620280	Early high-risk and locally advanced TNBC	Active, not recruiting
	Neoadjuvant chemotherapy with atezolizumab	NCT03281954	TNBC	Active, not recruiting
	Atezolizumab plus nab-paclitaxel	NCT04148911	Inoperable locally advanced or metastatic TNBC	Active, not recruiting
	Atezolizumab plus chemotherapy	NCT03371017	Inoperable recurrent TNBC	Recruiting
	Atezolizumab with adjuvant anthracycline/taxane-based chemotherapy	NCT03498716	Stage II-III TNBC	Recruiting
	Atezolizumab with ipatasertib and paclitaxel	NCT04177108	Inoperable locally advanced or metastatic TNBC	Active, not recruiting
Avelumab	Avelumab as adjuvant or postneoadjuvant treatment	NCT02926196	High-risk TNBC	Active, not recruiting
Camrelizumab	Camrelizumab plus chemotherapy	NCT04613674	Early or locally advanced TNBC	Recruiting
Serplulimab	Serplulimab combined with chemotherapy	NCT04301739	TNBC	Not yet recruiting
Toripalimab	Toripalimab combined with nab-paclitaxel	NCT04085276	Recurrent or metastatic TNBC	Recruiting
Carelizumab	Carelizumab combined with nab-paclitaxel and apatinib; carelizumab plus nab-paclitaxel; or nab-paclitaxel	NCT04335006	Inoperable locally advanced or metastatic TNBC	Recruiting
TQB2450	TQB2450 combined with anlotinib hydrochloride versus paclitaxel	NCT04405505	TNBC	Not yet recruiting
Adagloxad simolenin	Anti-Globo-H vaccine adagloxad simolenin (OBI-822)/OBI-821	NCT03562637	Early Globo-H^+^ TNBC	Recruiting

Although atezolizumab and pembrolizumab are the two leading mAbs for TNBC patients, other PD-1/PD-L1 mAbs have also been developed. For example, toripalimab (JS001, NCT04085276), HLX10 (NCT04301739), camrelizumab (NCT04613674), and avelumab (A-Brave, NCT02926196) have already entered phase III trials, and nivolumab (NCT04159818) and spartalizumab (NCT04802876) have been recruited for phase II trials. Due to the current limited immune response in the context of PD-1/PD-L1 targeting, researchers have begun to explore other targets, even double targets for ICB.

The role of chemokine receptor type 5 (CCR5) in modulating cell migration and the immune microenvironment is a potentially meaningful target in cancer. In the setting of cancer, increased CCR5 expression indicates a risk of tumor invasion and metastasis, and blocking CCR5 showed an exciting result in reducing tumor metastases by greater than 98% in a murine xenograft model [141]. Leronlimab (PRO140 targeting CCR5) initially received fast track FDA approval to treat human immunodeficiency virus infection. Currently, breakthrough therapy designation for leronlimab has been filed with the FDA to treat mTNBC [142]. An ongoing phase Ib/II clinical trial is being conducted to evaluate leronlimab in combination with carboplatin in CCR5-positive mTNBC, and preliminary analysis shows acceptable tolerability and efficacy [143]. The antibodies ipilimumab (NCT03546686) and tremelimumab (NCT02527434), which target CTLA4, have been assessed in TNBC. Lacnotuzumab (NCT02435680, targeting CSF1/MCSF), tigatuzumab (NCT01307891, targeting human death receptor 5), utomilumab (NCT02554812, targeting CD137), and LAG525 (NCT03499899, targeting lymphocyte activation gene-3) are actively being assessed phase II trials of TNBC.

However, ICB agents combined with conventional chemotherapeutic agents and small-molecular inhibitors have promoted their efficacy. Radiotherapy (NCT03004183) and other agents that promote immune initiation, such as tumor-associated vaccines (AE37 peptide vaccine in NCT04024800), oncolytic viruses (BT-001 in NCT04725331, talimogene laherparepvec in NCT04725331), and adenoviral-mediated IL-12 (NCT04095689), are being assessed in clinical phase II trials to enhance immunotherapy response. Collectively, ICB-related regimens plus other agents that might positively modify immunogenicity will be assessed in TNBC-related clinical trials soon.

### Bispecific antibodies

The clinical application of mAbs is fully warranted. However, cancer is a complex reticular disease involving multiple molecules, multiple steps, and complex mechanisms. Undeniably, certain benefits have been obtained by blocking or stimulating a specific target with mAbs, yet research on bispecific antibodies (BsAb) or bispecific T cell engager (BiTE) antibodies has progressed and evolved enormously over the past decades. BsAbs are engineered recombinant proteins designed to target two special antigens and theoretically have better antitumor effects. BiTE is a special BsAb that binds tumor-cell-specific antigen and cytotoxic T cells by binding and activating CD3 through a special engager to mediate cancer cells lysis. Catumaxomab (Removab, the EpCAM/CD3 BiTE) was the first BsAb approved by the FDA in 2009 followed by blinatumomab (Blincyto, the EpCAM/CD3 BiTE) in 2014 and emicizumab (Hemlibra) in 2017.

Bintrafusp alfa is a first-in-class bifunctional fusion protein that traps TGF-β and blocks PD-L1 in the tumor microenvironment. Regarding its structural design, the extracellular domain of the TGF-β RII receptor is fused with a human antibody against PD-L1. A phase I study of bintrafusp alfa reported a manageable safety profile, manageable tolerability, and preliminary efficacy in various solid tumors, especially in PD-L1 expressing tumors [144–146]. A submitted abstract of approximately 33 heavily pretreated TNBC patients from a phase I trial (NCT02517398) reported 1 case of CR, 2 cases of PR, 5 cases of disease control, with a median OS of 7.8 months [147]. A phase II trial of bintrafusp alfa is ongoing (NCT04489940).

The FDA recently considered a novel anti-EGFR/VEGFR2 BsAb [29]. Structurally, the BsAb includes the linker of the single-chain variable fragment of ramucirumab (VEGFR2 mAb) with the cetuximab (EGFR mAb) IgG backbone joined via a glycine linker. Therapeutically, the BsAb showed fair antitumor activity with multiple actions: inhibiting TNBC cell proliferation, attenuating the volume of the TNBC xenograft mouse model, impairing ligand-induced EGFR and VEGFR2 signaling, and preventing paracrine VEGFR2 signaling between endothelial cells and TNBC [29]. ADAM-17 is a matrix metalloproteinases-like proteases that is highly expressed in a variety of tumors and is an independent predictor of breast cancer prognosis. Previous studies found that an inhibitory mAb targeting ADAM-17 D1(A12) had anticancer activity in an in vitro model of TNBC [148]. A300E-BiTE targeting ADAM-17 also exhibited anticancer effects in prostate cancer cell lines [149], but the actual anti-TNBC effects of A300E-BiTE in TNBC must be explored further.

PD-L1 and CTLA4 are vital molecules suppressing T cell activation; hence, several BsAbs were designed for TNBC treatment, such as KN046 (NCT03872791, phase Ib/II), XmAb20717 (NCT03517488 phase I), and SI-B003 (NCT04606472, phase I). The BsAbs MGD013 (NCT03219268 and NCT04178460, both in phase I) and GEN1046 (NCT03917381, phase I/II) are all based on cotargeting by PD-L1 and lymphocyte activation gene-3 (LAG-3) and are also being tested in the clinic. Currently, engineered BsAbs are no longer limited to targeting tumor cells or tumor cells with T cells. BsAbs can target other inflammatory cells, such as natural killer (NK) cells or macrophages [150]. BsAbs can also target multiple activation signals simultaneously, such as DF1001 targeting NK and T cell activation signals to HER2-positive solid tumors (NCT04143711). Although most BsAbs are still in clinical trials for TNBC, they offer a potent approach.

### Antibody‒drug conjugates

The specific recognition between tumor cell antigens and antibodies can effectively direct the drug to tumor tissue rather than normal tissue. ADCs are new modified drugs that rely on four key components: cytotoxic drugs, a linker moiety, a humanized monoclonal antibody specifically recognizing neoplastic epitopes on cells, and overexpressed target antigens, such as HER2, trophoblast cell-surface antigen 2 (Trop-2), glycoprotein NMB (gpNMB) [151]. After recognizing the targeted antigens, the entire ADC molecule is degraded, or the linker is hydrolyzed due to specific features of the extracellular or intracellular microenvironment (i.e., a low pH in the high metabolic tumor microenvironment). ADCs offer a novel, personalized therapeutic approach with highly selective transport of agents. Sacituzumab govitecan was the only ADC approved by the FDA in 2020 for relapsed refractory mTNBC patients treated at least twice [123]. Glembatumumab vedotin and ladiratuzumab vedotin are new ADC drugs being assessed in clinical trials. Table 5 describes clinical trials of ADCs and their analogs in TNBC.

**Table 5:** Combination treatment for TNBC

Name/NCT number	Phase	Regimen	Cases	Patient cohort	Primary Endpoints (PFS, months; OS, months; RR, %; pCR, % and DFS, %)	Ref
NCT00080301, NCT00082433	III	Ixabepilone + capecitabine vs. capecitabine alone	443	Locally advanced or m TNBC	PFS: 4.2 vs. 1.7 m; OS: 9.0 vs. 10.4 m; RR: 31 vs. 15%	[22]
PrECOG 0105	II	Iniparib, gemcitabine and carboplatin	80	Early-stage TNBC and BRCA1/2 mutation-associated BC	pCR:36%	[25]
GeparSixto; GBG 66	II	Paclitaxel, doxorubicin, and bevacizumab with or without carboplatin	315	Untreated, nonmetastatic, stage II-III, TNBC and HER2^+^ BC	pCR: 53.2 vs 36.9%	[26]
GeparSixto	II	As above	291	As above	pCR in ITT: 56.8 vs. 41.4%; pCR in germline BRCA mutations: 65.4 vs. 66.7%; pCR in non-BRCA mutation: 55 vs. 36.4%; DFS in non-BRCA mutation: 85.3 vs. 73.5 m	[27]
NCT01216111	III	Paclitaxel + carboplatin vs. cyclophosphamide + epirubicin + fluorouracil + docetaxel	647	Operable TNBC after definitive surgery	DFS: 86.5 vs. 80.3%	[28]
CALGB 40,603 (Alliance)	III	Paclitaxel + doxorubicin + cyclophosphamide + carboplatin and/or bevacizumab	443	Stage II to III TNBC	Carboplatin with pCR breast: 60 vs. 44%; Bevacizumab with pCR breast 59 vs. 48%; Carboplatin with pCR breast/axilla: 54 vs. 41%	[29]
ChiCTR-TRC-14005019	II	Docetaxel, epirubicin with or without lobaplatin	125	Operable stage I to III TNBC	pCR breast: 93.5 vs. 73.0%	[30]
CBCSG006	III	Cisplatin plus gemcitabine vs paclitaxel plus gemcitabine	236	Untreated mTNBC	PFS: 7.73 vs. 6.47 m	[31]
I-SPY 2 TRIAL	II	Veliparib with carboplatin	72	stage II or III HER2^−^ BC	pCR: 51 vs. 26%	[34]
BrighTNess	III	Veliparib + carboplatin + paclitaxel vs. carboplatin + paclitaxel vs. paclitaxel	634	Stage II-III TNBC	pCR: 53.1 vs. 57.5 vs. 31.0%	[35]
NCT01837095	I	Eribulin and balixafortide	56	HER2^−^ mBC	PR: 30%	[86]
UCBG 12–1	II	Abiraterone acetate plus prednisone	34	AR^+^ metastatic or inoperable locally TNBC	CBR: 20.0%	[42]
NCT02971761	II	Enobosarm and pembrolizumab	16	AR^+^ mTNBC	CR: 6.3%; PR: 6.3%; SD: 12.5	[43]
BELLE-4	II/III	Paclitaxel with buparlisib or placebo	416	HER^−^ locally advanced or mBC without prior chemotherapy	PFS: 8.0 vs. 9.2 m; PFS in PI3K pathway-activated population: 9.1 vs. 9.2 m	[49]
LOTUS	II	Paclitaxel with ipatasertib or placebo	124	untreated inoperable, locally advanced or mTNBC	PFS: 6.2 vs. 4.9 m; PFS in PTEN-low: 6.2 vs. 3.7 m	[53]
FAIRLANE	II	Ipatasertib plus paclitaxel or placebo	151	Early TNBC	pCR: 17% vs. 13%; pCR with PTEN-low: 16% vs. 13%; pCR with altered PIK3CA/AKT1/PTEN 18% vs.12%	[54]
PAKT	II	Paclitaxel with capivasertib or placebo	140	Untreated m TNBC	PFS: 5.9 vs. 4.2 m; PFS with PIK3CA/AKT1/PTEN-altered: 9.3 vs. 3.7 m; OS: 19.1 vs. 12.6 m	[55]
NCT01931163	II	Everolimus plus cisplatin	22	Stage II/III TNB	RR: 22.7%	[56]
GeparQuinto	III	Anthracycline and taxane-containing chemotherapy, with or without bevacizumab	493	TNBC	Overall pCR: BRCA1/2 mutant 50%, no BRCA mutant 31.5%; Bevacizumab: BRCA1/2 mutant 61.5%, no BRCA mutant 35.6%	[64]
NCT03394287	II	Camrelizumab with continuous apatinib or intermittent apatinib	40	Advanced TNBC within three lines of systemic therapy	PFS: 3.7 vs. 1.9 m; objective RR: 43.3 vs. 0.0%; DCR: 63.3 vs. 40.0%	[66]
NCT02657889	II	Niraparib and pembrolizumab	47	advanced or metastatic TNBC	PFS: 8.3 m; ORR: 21.3%; objective RR in BRCA mutation: 46.6%	[36]
IMpassion130	III	Nab-paclitaxel plus atezolizumab or placebo	451	Untreated mTNBC	PFS: 7.2 vs. 5.5 m; PFS in PD-L1^+^: 7.5 vs. 5.0 m; OS: 21.3 vs. 17.6%; OS in PD-L1^+^: 25.0 vs. 15.5%	[134]
IMpassion131	III	Atezolizumab-paclitaxel	651	Untreated advanced TNBC	PFS in ITT: 5.7 vs. 5.6 m; PFS with PD-L1^+^: 6.0 vs. 5.7 m; OS in ITT: 14.2 vs. 14.5%; OS with PD-L1^+^: 15.2 vs. 15.8%; overall RR in ITT: 54 vs. 47%; overall RR with PD-L1^+^: 63 vs. 55%	[135]
IMpassion031	III	Nab-paclitaxel with atezolizumab or placebo	455	Untreated stage II-III TNBC	pCR in ITT: 57.6 vs. 41.1%; pCR with PD-L1^+^: 68.8 vs. 49.3%	[136]
KEYNOTE-355	III	Pembrolizumab + chemotherapy vs. placebo + chemotherapy	847	untreated locally recurrent inoperable or mTNBC	PFS in ITT: 7.5 vs. 5.6 m; PFS in CPS of 1 or more: 9.7 vs. 5.6 m	[137]
KEYNOTE-522	III	Pembrolizumab + paclitaxel + carboplatin vs. placebo + paclitaxel + carboplatin	602	untreated stage II or stage III TNBC	pCR: 64.8 vs. 51.2%	[138]

BRCA: breast cancer susceptibility gene; CBR: clinical benefit rate; CPS: combined positive score; CR: complete remission; DCR: disease control rate; DFS, disease-free survival; OS, overall survival; pCR, pathologic complete response; PFS, progression-free survival; PR, partial remission; RR, response rate; and SD: stable disease

The ADC sacituzumab govitecan-hziy (IMMU-132, trodelvy) consists of a humanized mAb targeting Trop-2 and an SN-38 payload (the active metabolite of irinotecan) joined via a cleavable CL2A linker. IMMU-132 could release both extracellular and intracellular SN-38. Extracellular SN-38 at therapeutic concentrations kills adjacent tumor cells, whereas internalized SN-38 kills bound tumor cells [152]. In a phase I/II study, 69 patients with mTNBC who had received a mean of five treatments were studied [153]. The CBR was 46% (including stable disease for ≥ 6 months), and the objective RR was 30% with PR accounting for 90% of the RR. The median OS was 16.6 m, and the median PFS was 6 m. Of note, neutralizing antibodies to IMMU-132 were not detected after repeated cycles, indicating that IMMU-132 has great prospects for application in early TNBC or combination therapy [154]. In another phase I/II single-group trial recruiting refractory advanced TNBC (2 to 10 previous anticancer regimens), 108 patients were treated with IMMU-132 [155]. Among these patients, 3 achieved CR, and 33 achieved PR. The median response duration was 7.7 months, the overall OS was 13.0 m, and the median PFS was 5.5 m. Remarkably, the median duration of treatment (5.1 months) was increased approximately two-fold compared with that of previous anticancer treatment (2.5 months).

Recently, the results of a phase III study of sacituzumab govitecan in 32 TNBC patients with metastases and recurrence within 12 months after neoadjuvant chemotherapy were reported. PFS and OS were longer in the sacituzumab govitecan-treated group compared with physician’s choice (eribulin, vinorelbine, gemcitabine, or capecitabine). The mean PFS was 5.7 (2.6–8.1) vs. 1.5 (1.4–2.6) months in the two groups; the mean OS was 10.9 (6.9–19.5) vs. 4.9 (3.1–7.1) months, respectively [156].

Another ADC, PF-06647020, which contains a humanized anti-protein tyrosine kinase 7 antibody that binds to the microtubule inhibitor auristatin-0101 via a cleavable valine–citrulline linker [157], showed a 21% ORR in a phase I trial (NCT02222922) enrolling 29 patients with TNBC [158]. A phase I trial of PF-06647020 plus gedatolisib for treating TNBC is underway (NCT03243331). Ladiratuzumab vedotin (SGN-LIV1A) is an ADC composed of a microtubule-disrupting agent and an antibody aimed at LIV-1, a multispan transmembrane protein. Preclinical data support that SGN-LIV1A is effective in mTNBC, and an ongoing clinical trial (NCT03310957) has evaluated the combination of SGN-LIV1A with pembrolizumab. EGFR and EpCAM are highly expressed in TNBC and thus are potential targets for ADC drugs. EpCAM- and EGFR-specific SNAP-tagged single-chain antibody fragments with monomethyl auristatin E show dose-dependent cytotoxicity in cell lines and could be promising ADCs for TNBC [159].

Earlier studies tested the good tolerability of glembatumumab vedotin (CDX-011) in locally advanced or metastatic BC and increased PFS in gpNMB-positive tumors [160, 161]. However, the results from the METRIC study (NCT01997333) for patients with gpNMB-positive mTNBC demonstrated no improvement PFS over capecitabine [162]. Mirvetuximab soravtansine, an ADC targeting folate receptor α, was terminated due to a low actual positive rate for folate receptor α (NCT03106077).

Other targets of ADCs such as MUC1 and the receptor d'origine nantais could also be further validated in TNBC [163, 164]. In addition, based on the specific binding of peptides to proteins, peptides represent the novel drug conjugates. For example, a peptide-docetaxel-conjugate (TH1902) targeting sortilin exerts anticancer effects in TNBC cells and tumor xenograft models [165]. A novel analog conjugate (CX-2009, praluzatamab ravtansine) was studied in 22 patients with advanced HR^+^/HER^−^ BC with the probody drug conjugate (PDC), showing that 9% patients had partial responses and 45% of patients had stable disease [166]. Praluzatamab ravtansine consists of anti-CD166 mAb, DM4, and a protease-cleavable linker covered with a shielding peptide. Therefore, praluzatamab is a novel ADC drug that is conditionally activated in a specific tumorous microenvironment (e.g., local high proteases) to avoid drug retention in normal tissue. PDC exhibits a revolutionary design for reducing the toxicity of ADCs, but its efficacy in TNBC must be further assessed.

Toxic drugs are common drugs loaded by ADCs. Several ADCs carrying immunomodulators, such as TAK-573, attenuated interferon alpha-2b and showed robust antitumor activity in nonclinical studies [167]. Therefore, the development of verified payloads able to transform the immune-suppressive microenvironment to an antitumor microenvironment in the body, disrupt cell signaling communication, or even change the adapted tumor metabolic microenvironment through alterations in oxygen supply, glycolysis, fatty acid, and amino acid metabolism may offer broad approaches for tumor treatment. However, several limitations of ADCs need to be improved further. For example, identification of the best binding antigen peptides, degradation pattern, and size are the key factors determining antibody development. In addition, increasing the payload antibody ratio and linking highly specific drugs to cancer cells are important issues that need to be solved. The toxic side effects of ADCs also require alarm; for example, the clinical development of PCA062 (an ADC targeting p-cadherin) was terminated given the high incidence of DM1 payload-related adverse events and limited antitumor activity [168]. SAR566658 (NCT02984683), an ADC targeting humanized DS6 (huDS6), was terminated in a phase II trial (NCT02984683) due to an ophthalmological event.

### Adoptive cell therapy (ACT)

T cell infiltration in TNBC is strongly correlated with prognosis, suggesting that adoptive cell therapy (ACT) offers a new therapeutic option for TNBC. ACT mainly includes CAR-T, T cell receptor therapy (TCR), and tumor-infiltrating lymphocyte therapy, and these methodologies all have similar principles. T cells from patients are expanded and genetically engineered in vitro to express synthetic TCRs or chimeric antigen receptors (CARs) that can target specific cancer antigens after reinfusion into the patient [169]. These infused cells trigger a cytotoxic immune response by recognizing tumor-associated antigens. This technology was initially applied to refractory hematologic malignancies. These engineered cells mainly involve T cells, but NK cells and CAR-M cells can also be engineered. Currently, CAR-T cells have been widely explored in solid tumors, but they are still in phase I clinical trials in TNBC (see Table 6).

**Table 6:** Clinical trials evaluating antibody–drug conjugates and analogues in patients with TNBC

Drugs	Category	Target	Payload	Register ID	Phase	Status
Sacituzumab govitecan	ADC	Trop-2	SN-38	NCT04468061	II	Recruiting
				NCT04454437	IIb	Active
				NCT04230109	II	Active
				NCT04595565	III	Recruiting
Datopotamab deruxtecan	ADC	Trop-2	Deruxtecan	NCT03401385	I	Recruiting
SKB264	ADC	Trop-2	Belotecan-derived payload	NCT04152499	I-II	Recruiting
Mirvetuximab soravtansine	ADC	Folate receptor α	DM4	NCT02996825	I	Active
				NCT03106077	II	Completed*
Ladiratuzumab vedotin	ADC	Zinc transporter LIV-1	MMAE	NCT03310957	Ib/II	Recruiting
NBE-002	ADC	ROR1	Anthracycline	NCT04441099	I/II	Recruiting
VLS-101	ADC	ROR1	MMAE	NCT04504916	II	Recruiting
BA3021	PDC	ROR2	Unpublished	NCT03504488	I/II	Recruiting
Camidanlumab tesirine	ADC	CD25	Pyrrolobenzodiazepine	NCT03621982	Ib	Recruiting
Praluzatamab ravtansine	PDC	CD166	DM4	NCT04596150	II	Recruiting
MGC018	ADC	CD276	Duocarmycin	NCT03729596	I/II	Recruiting
Anti-EGFR-IL-dox	Immunoliposomes	EGFR	Doxorubicin	NCT02833766	II	Unpublished
Trastuzumab deruxtecan	ADC	HER2	Deruxtecan	NCT04556773	Ib	Recruiting
Patritumab deruxtecan	ADC	HER3	Deruxtecan	NCT04699630	II	Recruiting
Anetumab ravtansine	ADC	Mesothelin	DM4	NCT03102320	Ib	Unpublished
				NCT02485119	I	Unpublished
Cofetuzumab pelidotin	ADC	Protein tyrosine kinase 7	Aur0101	NCT03243331	I	Unpublished
Enfortumab vedotin	ADC	Nectin-4	MMAE	NCT04225117	II	Recruiting
BT8009	Peptide drug conjugate	Nectin-4	MMAE	NCT04561362	I/II	Recruiting
TH1902 peptide	Peptide drug conjugate	Sortilin	Docetaxel	NCT04706962	I	Recruiting

DM4: tubulin-disrupting maytansinoid DM4; EGFR: epidermal growth factor receptor; gpNMB: glycoprotein nonmetastatic melanoma protein B; HER: human epidermal growth factor receptor; HR: Hormone receptor; MMAE: monomethyl auristatin E; ROR: receptor tyrosine kinase-like orphan receptor; SN-38: topoisomerase I inhibitor 7-ethyl-10-hydroxycamptothecin; TNBC: triple-negative breast cancer; and Trop-2: human trophoblast cell-surface antigen 2

*Failed to enroll enough patients

Since the positive clinical outcome of Kymriah and Yescarta, the era of ACT was opened, and CAR-T cells have been updated to the fifth generation. However, the treatment of ACT in TNBC is still under preliminary exploration. According to aberrantly expressed molecules in TNBC, CAR-Ts targeting c-MET (mRNA c-Met-CAR-T) [170], EphA10 (EphA10-specific CAR-T) [171], EGFR (EGFR CAR-T) [172], disialoganglioside GD2 (GD2-targeted CAR-T) [173], intercellular adhesion molecule-1 (ICAM1-specific CAR-T) [174], and mesothelin (CART-meso cells, NCT02580747) have already demonstrated antitumor efficacy in animal models, and some of these CAR-Ts have also been tested in clinical trials. Among the few reported CAR-T treatments for TNBC, four TNBC patients were treated with mRNA c-Met-CAR-T [170]. Two cases of disease death and two cases of disease progression were reported, and this agent failed to meet expectations. Hence, improving the effective intratumor transport of engineered activated T cells, preventing intratumor immunosuppressive signals, overcoming tumors heterogeneity, identifying tumor-specific antigens rather than tumor-associated antigens, and reducing the adverse effects of cell lysis from immune overactivation are issues that need to be addressed.

Several limitations of immunotherapy remain, but the methodology provides considerable medical promise. One of the most important aspects is the unsatisfactory RR to the current ICB regimens. Another concern is that there are no valid indicators to predict the effect of immunotherapy. The response to immunotherapy is influenced by multiple factors in the tumor immune microenvironment, not merely by target abundance. In addition to TILs, tumor-associated macrophages (TAMs) play a nonneglected immunosuppressive role in the tumor immune microenvironment. Preventing of the recruitment of TAMs, suppressing of the activation of M2 TAMs, switching the M2 phenotype to the antitumor M1 subtype, depleting of the number of immunosuppressive cells, and neutralizing inhibitory chemokines are strategies to reshape the tumor immune microenvironment [175]. Regardless, improving the survival rate is currently a priority for anticancer treatments. Other ICB regimens that are implemented for solid tumors, such as camrelizumab, should be accelerated only if these regimens offer better effects for patients with TNBC. Overcoming immune defects, enhancing the immune response, controlling adverse immune reactions and appropriate combinations will be addressed in future research.

## Combination therapies in TNBC

**Fig. 3:**
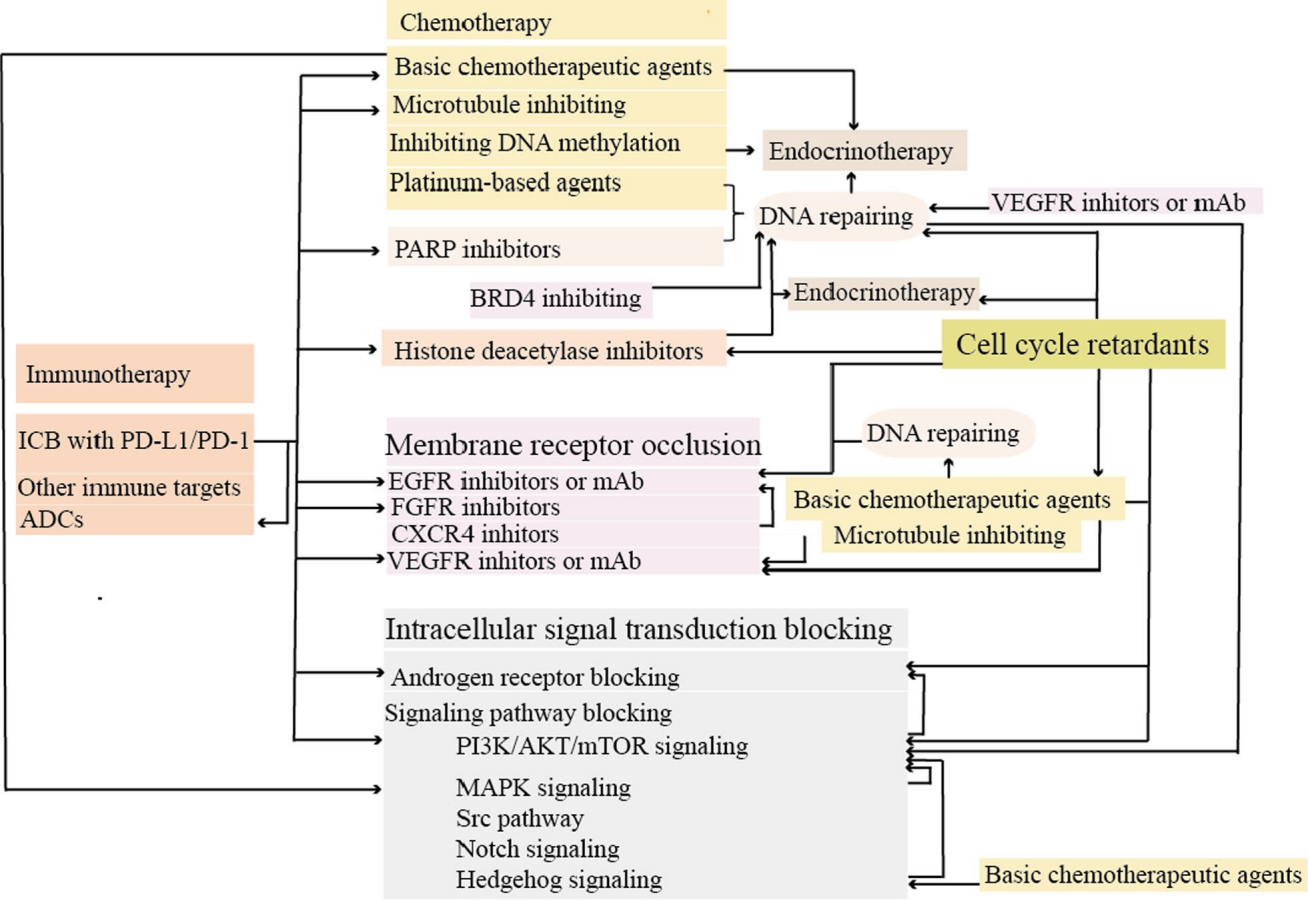
Summary of current combinations for TNBC treatment in clinical trials. The therapeutic strategies include immunotherapy and various molecular targeted therapies, including intracellular pathway inhibitors, cell cycle inhibitors, and AR inhibitors. ADCs: antibody‒drug conjugates; BRD4: bromodomain containing 4; ICB: immune checkpoint blockade; mAb: monoclonal antibody; and PARP: poly-adenosine diphosphate ribose polymerase

From the results of current TNBC clinical trials, the benefit of a single conventional anticancer therapy or immunotherapy is not sufficient due to tumor heterogeneity, tumor evolution and drug resistance. Therefore, combination therapy is currently the preferred option for TNBC treatment, and we summarize the primary endpoints of clinical trials in Table 7 and the current drug combination trials for TNBC in Fig. 3. From these studies, patients with nonadvanced TNBC had good responses after combination therapy; however, the prognosis of advanced TNBC still remained poor. Among them, PD-L1^+^ patients treated with conventional chemotherapy combined with immunotherapy as first-line therapy had a good prognosis [138], as described in the Sect. 4.2. In addition, those patients with BRCA-associated mutations have achieved a better prognosis after combination targeted therapy [64]. In second-line treatment, the novel ADC drug sacituzumab govitecan has been demonstrated undeniable effects [153, 154], and the combination therapy of ADC is worth exploring and looking forward to. Undoubtedly, precise personalized treatment of TNBC relies on the study of molecular expression characteristics and tumor biological mechanisms. Therefore, routine immunomolecular expression assessment and mutation analysis of TNBC tumor tissues are recommended, which will provide solid evidence for determining TNBC combination therapy regimens.

**Table 7:** Clinical trials evaluating adoptive cell therapy in patients with TNBC

Intervention	Register ID	Study population	Phase	Status
EGFR/ CD276	NCT05341492	EGFR/B7H3-positive advanced TNBC	I	Recruiting
ROR1-targeted CAR T cell (LYL797)	NCT05274451	ROR1 + relapsed or refractory TNBC	I	Recruiting
NKG2DL-targeting CAR-grafted gamma delta (γδ) T Cells	NCT04107142	Relapsed or refractory solid tumor	I	Unknown
c-Met RNA CAR T cells	NCT01837602	Metastatic breast cancer	0	Completed[170]
CART-TnMUC1 cells	NCT04025216	Advanced TnMUC1^+^ TNBC	I	Recruiting
Anti-meso-CAR vector transduced T cells	NCT02580747	Relapsed or chemotherapy refractory advanced TNBC	I	Recruiting
Mesothelin-specific chimeric antigen receptor-positive T Cells	NCT02792114	Metastatic HER2^−^ breast cancer	I	Active, not recruiting
PD-1^+^ TILS	NCT05451784	Advanced or metastatic TNBC	I/II	Not yet recruiting
TC-510	NCT05451849	Advanced mesothelin-expressing Cancer	I/II	Recruiting

CAR: chimeric antigen receptors; EGFR: epidermal growth factor receptor; HER: human epidermal growth factor receptor; ROR: receptor tyrosine kinase-like orphan receptor; TIL: tumor-infiltrating lymphocytes; and TNBC: triple-negative breast cancer

## Conclusions and perspectives

Triple-negative breast cancer remains a challenging subtype of breast cancer with a poor prognosis, and currently available treatments are not sufficient to address unresectable or recurrent TNBC tumors. In recent decades, knowledge on TNBC has increased significantly with the development of sequencing technologies and the emergence of new drugs, which are continuously updated in clinical trials. In this review, we summarize recent advances to solve the heterogeneity TNBC based on clinical and preclinical researches in the era of molecular cancer therapy and immunotherapy. At present, breakthroughs have been made in targeting homologous recombination defects as a new and effective therapeutic measure for TNBC, with platinum analogs and PARP inhibitors achieving considerable results even in the metastatic setting. In androgen receptor-positive TNBC, AR inhibitors and key enzyme inhibitors have improved clinical benefit rates. In addition, kinase inhibitors have also shown good promise.

Immunotherapy is an inevitable trend for the treatment of TNBC based on its characteristics. Immune checkpoint blockade by atezolizumab or pembrolizumab has offered partial benefits to patients. BsAb and BiTE have been developed to compensate for the unsatisfactory immune response rate of single-target monoclonal antibodies. In addition, novel ADCs are emerging, among which sacituzumab govitecan has been approved by the FDA. The application of ACT in solid tumors is under rapid development and, however, has not yet achieved good efficacy. Immunotherapy is now making important advances in the treatment of TNBC, but immunotherapy alone is not sufficient to treat TNBC, so combination therapy involving immunotherapy may be a better option to improve the outcome of TNBC.

To achieve better efficacy in the treatment of TNBC, the following aspects require continued research. First, more research is needed to improve the efficacy of existing drugs and to overcome drug resistance. Second, in some clinical trials, combination therapy has shown better efficacy than single drugs; however, the sequence and timing of combination drugs still require further study. Third, more research is needed to identify new targets, new biomarkers, and new drugs. We believe that with further advances in targeted therapeutic strategies for TNBC, patients with TNBC will have the opportunity to achieve better clinical outcomes.

In this review, we describe the subtypes and characteristics of TNBC, summarize recent advances in targeted therapy and immunotherapy, and discuss future directions to improve the clinical outcome of TNBC treatment. This scope of research will contribute to the development of precise individualized treatment of TNBC.

## Data Availability

Not applicable.

## Abbreviations

ACT: Adoptive cell therapy
ADCs: Antibody‒drug conjugates
AR: Androgen receptor
AXL: AXL receptor tyrosine kinase
BC: Breast cancer
BiTE: Bispecific T cell engager
BL: Basal-like
BLIS: Basal-like immune-suppressed
BRCA: Breast cancer susceptibility gene
BsAb: Bispecific antibody
CAR: Chimeric antigen receptors
CAR-T: Chimeric antigen receptor T
CBR: Clinical benefit rate
CCR5: Chemokine receptor type 5
CDKs: Cyclin-dependent kinases
CEF-T: Cyclophosphamide, epirubicin, and fluorouracil followed by docetaxel
CI: Confidence interval
CPS: Combined positive score
CSCs: Cancer stem cells
CTLA4: Cytotoxic lymphocyte antigen
CXCR4: C-X-C chemokine receptor type 4
CYP17A1: Cytochrome P450 family 17 subfamily A, polypeptide 1
DFS: Disease-free survival
DV1: CXCR4-binding peptide
EGFR: Epidermal growth factor receptor
ER: Estrogen receptor
FDA: The Food and Drug Administration
FGFR: Fibroblast growth factor receptor
gpNMB: Glycoprotein NMB
Hsp90: Heat shock protein 90
HDAC: Histone deacetylase
HER2: Human epidermal growth factor receptor 2
HR: Hormone receptor
HRD: Homologous recombination deficiency
IAPs: Inhibitors of apoptosis proteins
ICAM1: Intercellular adhesion molecule-1
ICB: Immune checkpoint blockade
IM: Immunomodulatory
iTILs: Intraepithelial TILs
LAG-3: Lymphocyte activation gene-3
LAR: Luminal androgen receptor
JAK2: Janus protein tyrosine kinase 2
KDM4B: Histone demethylase lysine demethylase 4B
M: Mesenchymal
mAb: Monoclonal antibody
MAPK: RAS/mitogen-activated protein kinase
mBC: Metastatic breast cancer
mTNBC: Metastatic triple-negative breast cancer
mTOR: Mammalian target of rapamycin
MSL: Mesenchymal-stem-like
NAC: Neoadjuvant chemotherapy
NK: Natural killer
ORR: Overall response rate
OS: Overall survival
PARP: The poly-adenosine diphosphate ribose polymerase
PARPi: The poly-adenosine diphosphate ribose polymerase inhibitors
PCb: Paclitaxel plus carboplatin
pCR: Pathological complete response
PD-1: Programmed cell death protein-1
PDC: Probody drug conjugate
PD-L1: Programmed death-ligand 1
PDX: Patient-derived tumor xenograft
PEG: Polyethylene glycol
PFS: Progression-free survival
PI3K: The phosphoinositol-3 kinase
PKB: Protein kinase B, also called AKT
PR: Progesterone receptor
PTEN: Phosphatase and tensin homolog
Rb: Retinoblastoma
RCB: Residual cancer burden
RR: Response rate
SMAC: Activator of caspases
sTILs: Stromal TILs
TAMs: Tumor-associated macrophages
TCGA: The Cancer Genome Atlas
TCR: T cell receptor therapy
]TILs: Tumor-infiltrating lymphocytes
TKIs: Tyrosine kinase inhibitors
TNBC: Triple-negative breast cancer
Trop-2: Trophoblast cell-surface antigen 2
VEGF: Vascular endothelial growth factor
VEGFR: Vascular endothelial growth factor receptor
